# Powering sustainable development within planetary boundaries†

**DOI:** 10.1039/c8ee03423k

**Published:** 2019-01-24

**Authors:** Ibrahim M. Algunaibet, Carlos Pozo, Ángel Galán-Martín, Mark A. J. Huijbregts, Niall Mac Dowell, Gonzalo Guillén-Gosálbez

**Affiliations:** Centre for Process Systems Engineering, Department of Chemical Engineering, Imperial College London South Kensington Campus London SW7 2AZ UK; Department of Environmental Science, Institute for Water and Wetland Research, Radboud University P.O. Box 9010 NL-6500, GL Nijmegen The Netherlands; Centre for Environmental Policy, Imperial College London, South Kensington Campus London SW7 1NA UK; Institute for Chemical and Bioengineering, Department of Chemistry and Applied Biosciences, ETH Zürich Vladimir-Prelog-Weg 1 8093 Zürich Switzerland gonzalo.guillen.gosalbez1@chem.ethz.ch

## Abstract

The concept of planetary boundaries identifies a safe space for humanity. Current energy systems are primarily designed with a focus on total cost minimization and bounds on greenhouse gas emissions. Omitting planetary boundaries in energy systems design can lead to energy mixes unable to power our sustainable development. To overcome this conceptual limitation, we here incorporate planetary boundaries into energy systems models, explicitly linking energy generation with the Earth's ecological limits. Taking the United States as a testbed, we found that the least cost energy mix that would meet the Paris Agreement 2 degrees Celsius target still transgresses five out of eight planetary boundaries. It is possible to meet seven out of eight planetary boundaries concurrently by incurring a doubling of the cost compared to the least cost energy mix solution (1.3% of the United States gross domestic product in 2017). Due to the stringent downscaled planetary boundary on biogeochemical nitrogen flow, there is no energy mix in the United States capable of satisfying all planetary boundaries concurrently. Our work highlights the importance of considering planetary boundaries in energy systems design and paves the way for further research on how to effectively accomplish such integration in energy related studies.

## Abstract 2

Designing energy systems within planetary boundaries is crucial to preserving the Earth's ecological capacity given the power sector's environmental footprint.

## Graphical abstract

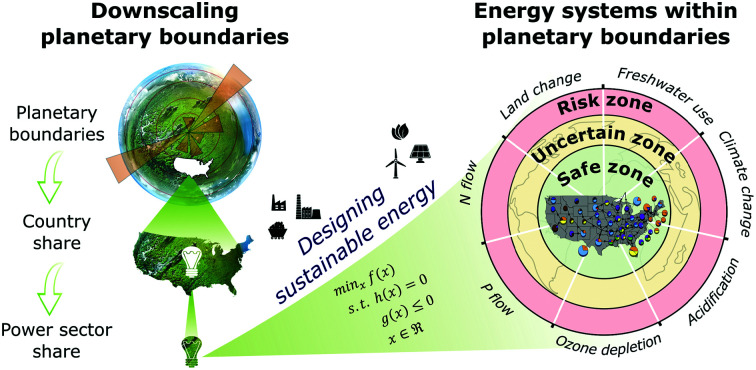

**Broader context**

Planetary boundaries are global limits on environmental flows that should never be transgressed to prevent the occurrence of catastrophic nonlinear events challenging the Earth's ecological capacity. Motivated by the planetary boundaries concept, the World Business Council for Sustainable Development created a clear sustainable pathway known as Action2020, which reflects upon the global momentum towards a broader scope of environmental policies going beyond greenhouse gas emissions. The power sector is key to sustainability, yet it has never been studied through the lens of planetary boundaries. Energy systems are intrinsically complex due to the wide range of engineering and reliability constraints governing their behavior. Developing decision-support tools for designing energy systems meeting these constraints while operating within planetary boundaries is considered critical. Here, we provide an approach that (i) characterizes environmental flows of electricity technologies in terms of planetary boundaries contributions, (ii) downscales planetary boundaries to the regional and sectoral level and (iii) incorporates both pieces of information into energy systems models to design more sustainable energy mixes. Our approach can assist in designing energy mixes that meet planetary boundaries, while opening up new avenues for the widespread incorporation of planetary boundaries in energy related problems.

## Introduction

Designing sustainable energy mixes of the future is a complex task that requires the use of energy systems models (ESMs) to support decision-making. ESMs available at present identify electricity mixes that minimize the total cost while meeting a set of technical constraints, including demand satisfaction and capacity limitations. MARKAL/TIMES,^1^ NEMS,^2^ SWITCH^3,4^ and Electricity Systems Optimization (ESO)^5^ are examples of ESMs that follow the same mathematical principles and general approach while differing in the modeling assumptions and data sources.

In past years, it has become clear that reducing Greenhouse Gas (GHG) emissions^6–10^ is not sufficient to ensure our sustainable development, since other key environmental impacts cannot be overlooked (*e.g.*, land-system changes or freshwater use). Hence, ESMs face at present the challenge of embracing additional environmental criteria beyond cost minimization to guide us towards sustainable energy systems. Recent works incorporated environmental impacts in ESMs following primarily Life Cycle Assessment (LCA) principles.^11–14^ Unfortunately, while enabling a deeper environmental analysis, the integration of LCA with ESMs fails to provide absolute bounds on the impact of an energy mix and, consequently, cannot identify sustainable energy mixes guaranteed to operate within the Earth's ecological capacity.^15^ Here, we present an approach to assist in the design of sustainable energy mixes based on the concept of Planetary Boundaries (PBs),^16^ a set of ecological limits that should never be transgressed by our planet to operate safely. Originally introduced by Rockström *et al.*,^17^ PBs on nine out of ten Earth-system Processes (ESPs) identify thresholds for humanity that, if surpassed, may trigger a series of nonlinear changes with unpredictable effects at a global scale.^18^ By including PBs in ESMs, we here link unambiguously the performance of electricity generation technologies with their wide environmental impact, ultimately designing energy mixes fully consistent with the Sustainable Development Goals.^19,20^

To demonstrate the capabilities of our approach, the United States (US) was taken as a testbed to discuss the implications of including PBs in energy systems design. Our previously developed ESM for the US, the Emissions Reduction Cooperation Model (ERCOM),^6^ was modified to include planetary boundaries (ERCOM with Planetary Boundaries (ERCOM-PB) henceforth) to ultimately identify mixes operating below all PBs and to analyze the economic implications thereof. We found that the Business as Usual (BAU) energy mix, *i.e.*, the US 2012 default developments in the power sector required to meet the 2030 electricity demand, transgresses six out of the eight PBs considered, including the climate change PBs. Furthermore, the least cost energy mix, which is in line with the 2 degrees Celsius (°C) target governed by the Paris Agreement (the Paris Agreement solution henceforth), transgresses five out of the eight PBs, including those on climate change, though in this solution the magnitude of the transgression is lower. Our model could not identify any single mix satisfying all the shares of PBs assigned to the US power sector concurrently, yet seven of the eight PBs could be met simultaneously with an energy mix relying on wind (both onshore and offshore), natural gas with Carbon Capture and Storage (CCS), hydropower and Bio-energy with CCS (BECCS). This mix would incur an extra cost of 40% compared to the BAU solution (or 0.8% of the US Gross Domestic Product (GDP) in 2017), while doubling the cost of the Paris Agreement solution (or an extra cost equal to 1.3% of the US GDP in 2017). This extra cost needed to improve the performance of the mix in terms of PBs would be associated with mandatory investments in relatively expensive electricity technologies, such as wind offshore, natural gas with CCS and BECCS.

## Modeling framework

**Fig. 1:**
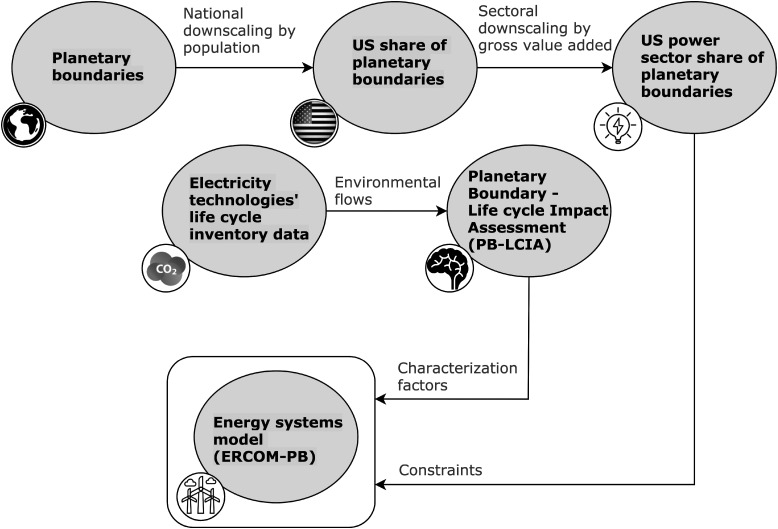
Modeling framework linking planetary boundaries with energy systems models. The PB-LCIA framework is based on published characterization factors that link life cycle inventories to Earth-system processes associated with planetary boundaries.

When attempting to incorporate PBs into ESMs, two main challenges were addressed (Fig. 1): (i) downscaling PBs, originally defined for the Earth as a whole, to the country and sectoral level; and (ii) linking the operation of energy technologies to their performance in terms of PBs *via* the environmental flows they generate. We discuss both challenges in detail next.

### Downscaling planetary boundaries to the sectoral level

PBs, defined at the global level, need to be downscaled first to the national and sectoral level to provide shares (*i.e.*, an upper bound on the total impact) that shall be respected by the US power sector in 2030 (Fig. 1). Allocating shares of the safe operating space defined by the concept of PBs among countries and sectors is a controversial step, as it requires applying sharing principles on which no general agreement has been reached so far (*e.g.*, sharing them equally among the world population^21–23^ or allocating them following tailored allocation procedures, such as expert judgment and past impacts^24–26^). Here, without loss of generality, we apply an egalitarian-based sharing principle,^27^ whereby the US power sector share of the safe operating space is quantified from the ratio of the US population to the global population times the ratio of the US power sector Gross Value Added (GVA) to the GVA of the whole US economy (Fig. 1) as follows:1
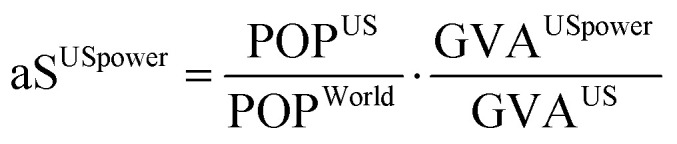
where aS^USpower^ is the share of the total safe operating space assigned to the US power sector, POP^US^ is the US population in 2016, POP^World^ is the world population in 2016, GVA^USpower^ is the GVA for the US power sector in 2016 and GVA^US^ is the GVA for the US total economy in 2016. Data sources for population and GVA are available in Table S1 (ESI†). Note the derived share, aS^USpower^, *via* the allocation procedure highlighted in eqn (1) is PB-independent.

The share of the safe operating space, eqn (1), is then multiplied by the full safe operating space for every PB, yielding environmental bounds that are imposed in ERCOM-PB to ensure that the mix sought will not transgress any key ecological limit:

2 SoSOS_*p*_ = aS^USpower^·SOS_*p*_ ∀*p*

where SoSOS_*p*_ is the US power sector absolute share of the safe operating space for every PB *p* and SOS_*p*_ is the full safe operating space for every PB *p*. The full safe operating space is a budget given by the difference between the PB and the natural background level, where the latter indicates the performance of each ESP before human intervention. Table 1 shows the PB, natural background level, full safe operating space and the US power sector share of the full safe operating space for the boundaries considered in this work. Eight out of fourteen global and regional PBs on seven out of ten ESPs were considered for the reasons discussed next.

**Table 1:** Planetary boundaries, natural background level, full safe operating space and assigned share of the full safe operating space to the US power sector obtained *via*eqn (2)

Planetary boundary title	Unit	Planetary boundary^18^	Natural background level^18,27^	Full safe operating space	Assigned share of safe operating space to the US power sector (aS_USpower_ = 0.062%)
Climate change (atmospheric CO_2_ concentration)	ppm CO_2_	350	278	72	0.045
Climate change (energy imbalance at top-of-atmosphere)	W m^−2^	1	0	1	6.19 × 10^−4^
Stratospheric ozone depletion^a^	DU	275	290	15	0.009
Ocean acidification^a^	Ω_arag_	2.75	3.44	0.69	4.27 × 10^−4^
Biogeochemical phosphorus flow (global)	Tg P year^−1^	11	1.1	9.9	0.006
Biogeochemical nitrogen flow (global)	Tg N year^−1^	62	0	62	0.038
Land-system change (global)^a^	%	75	100	25	0.015
Freshwater use (global)	km^3^ year^−1^	4000	0	4000	2.476

a Planetary boundaries on stratospheric ozone depletion, ocean acidification and land-system change act as lower bounds^18^ and hence when the full safe operating space is calculated, the absolute value should be considered.^27^

### Selection criteria for planetary boundaries in ERCOM-PB

Some PBs still show data and methodological gaps that prevent their full use in some practical application. Here, PBs relevant to the US energy systems were chosen based on (i) availability of the corresponding inventory entries in life cycle repositories; and (ii) applicability of the specific PB to our region of interest (*i.e.*, the US). Based on the first criterion, the biosphere integrity PBs and the regional PBs on land-system change, freshwater use and biogeochemical Phosphorus (P) flow were omitted. Based on the second criterion, we discarded the PB on atmospheric aerosol loading, as the current PB focuses on the Indian subcontinent.^18^ Similarly, the PB on the introduction of novel entities was also omitted because it has not been formally defined yet.^18^ This leads to the consideration of eight out of fourteen global and regional PBs on seven out of ten ESPs already identified in the literature.^18^

### Linking life cycle inventories to planetary boundaries

Understanding and identifying the drivers of ESPs is the key that paves the way for the inclusion of PBs into ESMs. ESPs may seem ramified and difficult to reconcile in a single modeling environment, but a closer look delineates roots to cluster them into a number of environmental flows (or life cycle inventories) that could potentially be harnessed in environmental policies. These life cycle inventories are connected to ESPs *via* characterization factors recently proposed in the literature by Ryberg *et al.*^28^ as follows:3
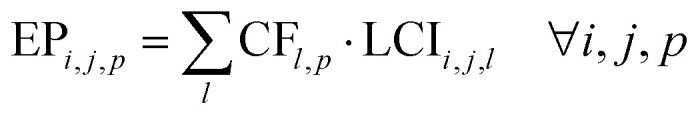
where EP_*i*,*j*,*p*_ is the total environmental burden generated per unit of energy supplied by electricity technology *i* in state *j* linked to PB *p*, CF_*l*,*p*_ is a characterization factor that links life cycle inventory entry *l* to PB *p* and LCI_*i*,*j*,*l*_ is the life cycle inventory *l* generated when one unit of electricity is supplied *via* technology *i* in state *j*. We next discuss the relevant life cycle inventories to each PB.

Two PBs, atmospheric CO_2_ concentration (ppm) and energy imbalance at top-of-atmosphere (W m^−2^), are used to describe climate change, which is responsible for both physical and biosphere fluctuations.^17,18^ GHG emissions such as CO_2_, CO, CH_4_ and NMVOC quantify the atmospheric CO_2_ concentration PB, while additional air emissions such as N_2_O, NF_3_, SF_6_, CFCs and HCFCs characterize in turn the energy imbalance PB.^28^

Stratospheric ozone depletion PB, measured in Dobson Unit (DU), evaluates the role of the ozone layer in filtering ultraviolet radiation from the Sun.^17^ Ozone depleting substances remove stratospheric ozone *via* reaction with chlorine and bromine;^29^ these include air emissions such as CFCs, HCFCs, Halons and R-40.^28^ Although the existing method to compute PBs^28^ does not assign a characterization factor that quantifies the N_2_O impact on the stratospheric ozone depletion PB, N_2_O emissions do exert a noticeable pressure on the ozone layer.^30^ Therefore, we here expand the existing method developed by Ryberg *et al.*^28^ by designing a characterization factor that quantifies the impact of N_2_O emissions on the stratospheric ozone depletion PB. In essence, we first convert the N_2_O emissions to CFC-11 equivalent (*i.e.*, 0.018 kg of CFC-11 equivalent per kg of N_2_O)^30^ and then apply the characterization factor that links CFC-11 to the stratospheric ozone depletion PB available in the existing framework.^28^

The ocean acidification PB is measured in Ω_arag_. Since marine species are vulnerable to changes in the CO_2_ chemistry of oceans,^17^ this PB is closely related to the atmospheric CO_2_ concentration PB. An increasing concentration of CO_2_ in the ocean could raise its acidity causing many aragonite shells to dissolve.^17^ Ocean acidification mangles marine biodiversity and, therefore, it could affect the ability of oceans to sink CO_2_.^17,18^ Similar to the atmospheric CO_2_ concentration PB, though using different characterization factors, the ocean acidification PB is quantified *via* air emissions that include CO_2_, CO, CH_4_ and NMVOC.^28^

Freshwater eutrophication is connected to PBs that limit global biogeochemical P and Nitrogen (N) flows, measured in Tg P and Tg N, respectively.^18^ An excess amount of biogeochemical P and N flows induced by human interventions could disturb their global cycles,^31,32^ pushing marine systems across their tipping point.^17^ Global biogeochemical P flow is quantified from the P flow to freshwater; meanwhile, the biogeochemical N flow is obtained from NO_*x*_ and NH_3_ to air, N-tot and NO_3_^−^ to freshwater and NO_3_^−^ to groundwater.^28^

Land-system change PB, expressed as a percentage of the original forest cover, evaluates the way in which climate is regulated through the exchange of energy and water between land and the atmosphere.^18^ The global PB on land-system change imposes a lower bound on the forest cover remaining area relative to the original forest cover.^18^ The land-system change PB is linked to the area of forest transformation associated with a given process.^28^

The freshwater use PB, measured in km^3^, limits the consumption of blue water.^18,33^ This PB reflects the vulnerability of biodiversity, food and health security to global manipulation of the freshwater cycle.^17^ When implementing this PB into an ESM, freshwater from rivers, lakes, reservoirs and groundwater should be considered to ensure consistency with the designed PB on freshwater use.^18,33^

To evaluate PBs, life cycle inventory entries, LCI_*i*,*j*,*l*_, connected to PBs were retrieved from the ecoinvent LCA database^34,35^ as well as from other specific technically sound sources^36–39^ (Table S1, ESI†). Published characterization factors, CF_*l*,*p*_, were applied to translate the life cycle inventories of electricity technologies into the environmental burdens, EP_*i*,*j*,*p*_, linked to PBs (eqn (3)).^28^

### The emissions reduction cooperation model with planetary boundaries

The PBs mentioned previously were incorporated into ERCOM,^6^ an ESM tailored to the US that minimizes the electricity cost for a given target on emissions, imposed either at the national or state level. The new model, referred to as ERCOM-PB, incorporates PBs and assumes full cooperation among states – that is, the US operates as a single entity to satisfy the domestic electricity demand while meeting a national environmental target. ERCOM-PB is formulated as a linear programming model that finds the least cost pathway (*i.e.*, electricity mix) minimizing the total transgression of downscaled PBs to the US power sector, while considering technical constraints relevant to energy mixes.

The model is outlined next, while a full description of ERCOM-PB and the corresponding data sources is available in Notes S1, S2 and Table S1 (ESI†). Furthermore, the uncertainty analysis approach is described in Note S3 (ESI†), while the main limitations of our modeling framework are summarized in Note S4 (ESI†).

ERCOM-PB can be expressed in compact form as follows:4
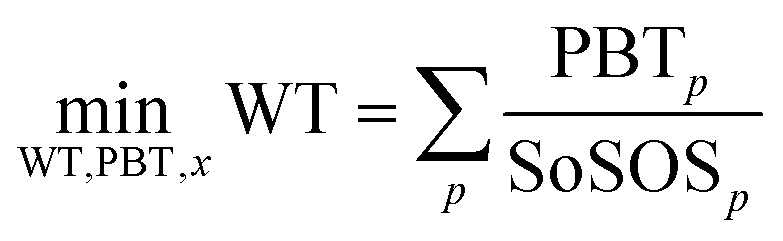
s.t.

5 PBT_*p*_ ≥ 0 ∀*p*

6
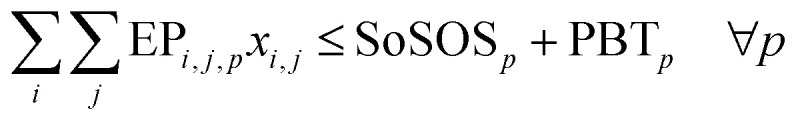

7 *A*_*i,j*_*x*_*i,j*_ ≤ *a*_*i,j*_ ∀*i,j*

8 WT, PBT, *x* ∈ 

where WT is a continuous variable that quantifies the value of the objective function to be minimized given by the summation of the weighted transgression magnitude of every downscaled PB *p* by the US power sector share of the safe operating space, PBT_*p*_ is a continuous positive variable that measures by how much each downscaled PB *p* is transgressed, EP_*i*,*j*,*p*_ is the total environmental burden generated per unit of energy supplied by each technology *i* in every state *j* linked to PB *p*, *x*_*i*,*j*_ is a continuous variable denoting the amount of electricity supplied in 2030 by technology *i* in state *j*, SoSOS_*p*_ is the US power sector absolute share of the safe operating space for every PB *p* derived *via*eqn (2), *A*_*i*,*j*_ is the engineering technical matrix of constraints defined for technology *i* in state *j*, *a*_*i*,*j*_ is the accompanying upper bound vector for each engineering constraint (*e.g.*, the generation potential of a technology limits its electricity generation) for technology *i* in state *j* and ℝ is the set of real numbers to which variables WT, PBT_*p*_ and *x*_*i*,*j*_ belong.

### Solutions definition

To underscore the importance of incorporating PBs in designing sustainable energy mixes, we analyze three electricity mixes that meet the expected electricity demand in 2030 (Table 2). The ‘BAU’ solution (S1) represents the US 2012 default developments in the power sector to meet the 2030 electricity demand (*i.e.*, the same breakdown of technologies as in 2012 would be implemented to cover the energy demand in 2030). The ‘Paris Agreement’ solution (S2) corresponds to the least cost solution that meets the US commitment to the Paris Agreement 2 °C target in 2030 while satisfying the same electricity demand as in the BAU solution. To obtain this mix, we solved a slight variant model of ERCOM-PB, where objective function (9) replaces objective function (4) and inequality (10) replaces inequalities (5) and (6) as follows:9
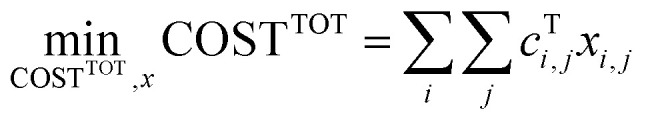
s.t.10
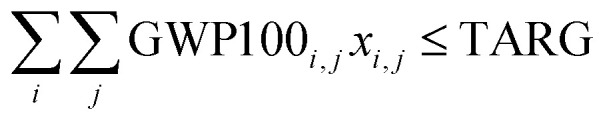

11 *A*_*i,j*_*x*_*i,j*_ ≤ *a*_*i,j*_ ∀*i,j*

12 COST^TOT^, *x* ∈ ℝ

where COST^TOT^ is a continuous variable that quantifies the value of the objective function to be minimized given by the summation of the total cost of electricity supply, *c*^T^_*i,j*_ is the Levelized Cost of Electricity (LCOE) of technology *i* in state *j*, GWP100_*i*,*j*_ is the 100-year Global Warming Potential (GWP100) per unit of energy generated by technology *i* in state *j* and TARG is the GWP100 target in line with the US commitment to the Paris Agreement 2 °C target in 2030. That is, PBs are not enforced, but the model needs to meet a bound on the GWP100 consistent with the Paris Agreement 2 °C target in 2030. As shall be discussed later, the model produces the same solution regardless of whether the GWP100 limit is enforced or not, that is, the minimum cost mix satisfies the Paris Agreement 2 °C target even when this condition is not included explicitly in the model. This is due to the economic competitiveness of some renewable technologies with very low carbon emissions (Table S2, ESI†).^40^

**Table 2:** Solutions definition

Solution title	Solution label	Description
Business as usual	S1	The 2012 energy mix in 2030, where the share of each technology is fixed to its 2012 level and the demand is projected to 2030
Paris Agreement	S2	The energy mix in 2030 that would meet the US commitment to the Paris Agreement 2 degrees Celsius target and the projected demand in 2030 at minimum cost
Planetary boundaries	S3	The energy mix in 2030 that would minimize the transgression of planetary boundaries at minimum cost

Finally, the ‘Planetary boundaries’ solution (S3) is obtained as follows. We first solve ERCOM-PB (4)–(8) to find the solution with the smallest transgression of PBs. As cost is not accounted for in the objective function, the model could find solutions with the same level of transgression but less costly that would be, therefore, more appealing for decision-makers. Hence, in a subsequent step, we fix the amount by which PBs are transgressed in the optimal solution of ERCOM-PB (*i.e.*, the minimum transgression possible among all the electricity mixes satisfying the technical constraints and meeting the demand) and minimize the total cost (eqn (9)), finding solution S3.

## Results and discussion

### Myopic policies to planetary boundaries

**Fig. 2:**
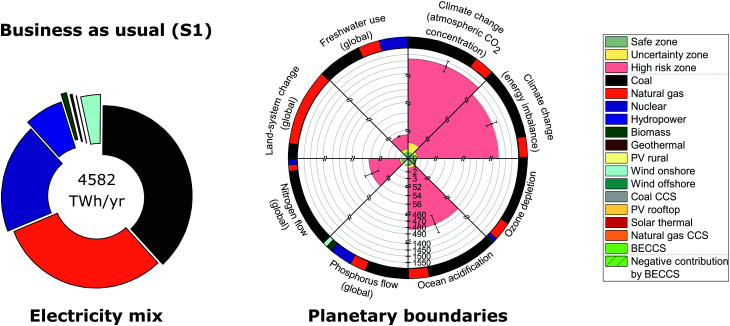
Global US electricity generation and imports portfolio and performance relative to downscaled planetary boundaries for the business as usual energy mix (solution S1). The circle on the left represents the 2012 energy mix in 2030; its performance in terms of planetary boundaries is summarized in a rose chart on the right. The rose chart represents the current performance of each Earth-system process divided by the US power sector share of the full safe operating space (*i.e.*, a value less than one implies it lies below the planetary boundary). The green zone is the safe operating space (*i.e.*, within the strictest planetary boundary in the uncertainty range), the yellow zone is the uncertainty range of each planetary boundary and the red zone is beyond the least strict planetary boundary in the uncertainty range. Each error bar corresponds to one standard deviation considering the uncertainty in environmental burdens (*i.e.*, life cycle inventory entries) connected to planetary boundaries. Each circular sector of the outer ring on the right represents the intensity of each technology on each planetary boundary. The labeling of solutions is given in Table 2.

We start by analyzing the US electricity mix in the BAU solution (S1) through the lens of PBs (Fig. 2). We assumed the most stringent PBs values as suggested by Steffen *et al.*^18^ and considered the main uncertainties associated with the environmental burdens (*i.e.*, life cycle inventory entries) connected to PBs as well as with the LCOE values of electricity technologies (Note S3, ESI†). The results of the uncertainty analysis of the environmental burdens are reported in the figures as error bars, where each error bar represents one standard deviation.

We found that six out of the eight PBs considered are transgressed in solution (S1), including atmospheric CO_2_ concentration, energy imbalance at top-of-atmosphere, ocean acidification, biogeochemical N flow, freshwater use and stratospheric ozone depletion (Fig. 2), which raises significant concerns about our future ability to deliver sustainable energy without altering the current status quo. Only two PBs would hence be satisfied, namely biogeochemical P flow and land-system change.

Though the transgression of the PB on stratospheric ozone depletion is within the uncertainty zone (*i.e.*, the yellow zone in Fig. 2), the remaining transgressed PBs in the BAU mix fall beyond the uncertainty zone (*i.e.*, the red high risk zone in Fig. 2), indicating there is certainty in failing to operate within the Earth's safe operating space. PBs on climate change are transgressed the most; the PB on atmospheric CO_2_ concentration is transgressed by 1515 times above the limit, while energy imbalance at top-of-atmosphere is transgressed by 1442 times above the limit. They are followed by ocean acidification, transgressed by 483 times above the limit. Analyzing the contribution of the electricity technologies to PBs, we found that the deployment of coal and natural gas power plants seem to be the main reason behind the transgression of the climate change PBs and the PB on ocean acidification (Fig. 2). At a lesser transgression extent, the global PB on biogeochemical N flow is surpassed by 55 times above the limit, mainly due to the reliance on coal power plants. Moreover, freshwater use transgresses the limit by fourfold due to the deployment of coal, nuclear and natural gas power plants. Finally, stratospheric ozone depletion is transgressed the least (*i.e.*, 18% above the limit) mainly due to the reliance on coal power plants and, albeit to a lesser extent, natural gas plants.

**Fig. 3:**
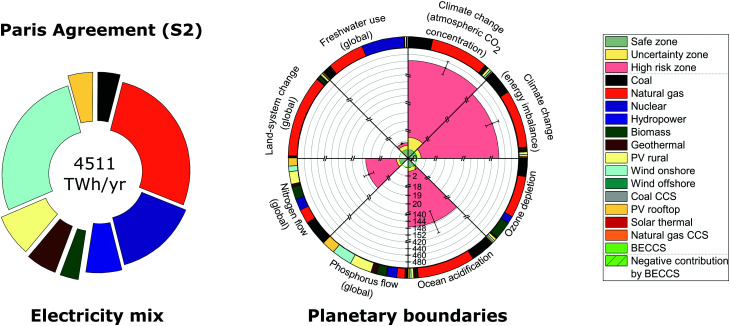
Global US electricity generation and imports portfolio and performance relative to downscaled planetary boundaries for the Paris Agreement mix (solution S2). The circle on the left represents the energy mix in 2030 that meets the Paris Agreement 2 degrees Celsius target at minimum cost; its performance in terms of planetary boundaries is summarized in a rose chart on the right. The rose chart represents the current performance of each Earth-system process divided by the US power sector share of the full safe operating space (*i.e.*, a value less than one implies it lies below the planetary boundary). The green zone is the safe operating space (*i.e.*, within the strictest planetary boundary in the uncertainty range), the yellow zone is the uncertainty range of each planetary boundary and the red zone is beyond the least strict planetary boundary in the uncertainty range. Each error bar corresponds to one standard deviation considering the uncertainty in environmental burdens (*i.e.*, life cycle inventory entries) connected to planetary boundaries. Each circular sector of the outer ring on the right represents the intensity of each technology on each planetary boundary. The labeling of solutions is given in Table 2.

We next analyze solution S2, that is, the grid that would emerge from the cost minimization of the US mix considering a limit on GHG emissions; to this end, we used the GWP100 in line with the US Intended Nationally Determined Contribution (INDC) under the Paris Agreement 2 °C target. The US INDC commits the nation to reduce its GWP100 by 26–28% in 2025 (or around 39% in 2030) compared to the 2005 levels.^41^ Note that due to electricity transmission losses between neighboring regions, the electricity generated in solution S2 differs from that in solution S1. In terms of the mix that would emerge in solution S2, we found that the Paris Agreement mix would displace primarily coal power plants by renewables, particularly wind onshore (Fig. 3). In fact, even after enforcing the Paris Agreement target (S2), only one additional PB, namely the one on stratospheric ozone depletion, is met compared to the BAU mix (S1) due to the displacement of coal power plants – five out of the eight PBs considered would still be transgressed. The transgressed PBs in solution S2 include both PBs on climate change, as well as PBs on ocean acidification, biogeochemical N flow and freshwater use.

The Paris Agreement mix (S2) would reduce the transgression of every PB ranging from 49 to 69% in contrast to the BAU mix (S1); however, the five transgressed PBs would still lie in the PBs high risk zone (the red zone in Fig. 3). In fact, both PBs on atmospheric CO_2_ concentration and energy imbalance at top-of-atmosphere are transgressed by 462 and 442 times above the limit, respectively, as both PBs on climate change are more stringent than the Paris Agreement 2 °C target. The PB on ocean acidification is also transgressed by 147 times above the limit. Similar to the BAU mix, the reliance of solution S2 on natural gas and, to a lesser extent, coal power plants contribute to the transgression of the climate change PBs and the PB on ocean acidification (Fig. 3). Furthermore, solution S2 transgresses the PB on biogeochemical N flow by 20 times above the limit due to the deployment of coal, biomass and PV plants. Lastly, the freshwater use PB is transgressed by two times above the limit due to the deployment of nuclear, coal and natural gas power plants.

### Minimizing the transgression of planetary boundaries

**Fig. 4:**
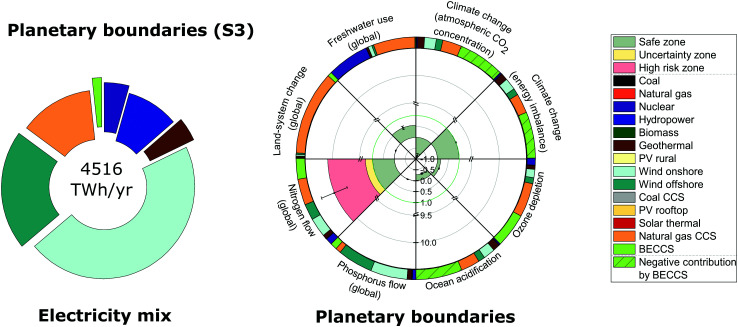
Global US electricity generation and imports portfolio and performance relative to downscaled planetary boundaries for the planetary boundaries mix (solution S3). The circle on the left represents the energy mix in 2030 that would minimize the transgression of planetary boundaries at minimum cost; its performance in terms of planetary boundaries is shown in a rose chart on the right. The rose chart represents the current performance of each Earth-system process divided by the US power sector share of the full safe operating space (*i.e.*, a value less than one implies it lies below the planetary boundary). The green zone is the safe operating space (*i.e.*, within the strictest planetary boundary in the uncertainty range), the yellow zone is the uncertainty range of each planetary boundary and the red zone is beyond the least strict planetary boundary in the uncertainty range. Each error bar corresponds to one standard deviation considering the uncertainty in environmental burdens (*i.e.*, life cycle inventory entries) connected to planetary boundaries. Each circular sector of the outer ring on the right represents the intensity of each technology on each planetary boundary. The labeling of solutions is given in Table 2.

ERCOM-PB is next solved aiming to satisfy all PBs simultaneously (solution S3 shown in Fig. 4). We found that no energy mix exists that can meet all of the PBs concurrently. More precisely, no single energy mix can satisfy the global biogeochemical N flow PB, noting that in this PB, solution S3 lies in the high risk zone (red area in Fig. 4).

The mix in solution S3 reduces the number of coal and natural gas plants while deploying BECCS, wind onshore and offshore and natural gas with CCS power plants; this allows the mix to meet both PBs on climate change as well as the PB on ocean acidification (Fig. 4). Overall, solution S3 shows a net negative contribution towards both PBs on atmospheric CO_2_ concentration and ocean acidification, mainly due to the deployment of BECCS, a net negative emission technology on a life cycle basis.^39^ On the other hand, the energy imbalance PB assigns larger weights to CFCs and other GHG emissions compared to CO_2_; consequently, BECCS shows a lower net negative contribution in this category (Note S5 and Fig. S1, ESI†). Overall, the deployment of low (or negative) GHG emissions technologies (on a life cycle basis) allows the mix to meet the PB on energy imbalance at top-of-atmosphere.

Solution S3 meets the freshwater use PB by partially displacing natural gas and nuclear plants while completely phasing out coal plants. Furthermore, the deployment of hydropower and wind onshore and offshore plants reduces the transgression of the global biogeochemical N flow PB by 49% compared to the Paris Agreement mix (S2) and 81% compared to the BAU mix (S1). In solution S3, this transgression is mainly due to the deployment of BECCS and natural gas with CCS plants as well as the presence of nuclear plants.

The contribution of major environmental flows (*i.e.*, life cycle inventories) towards the performance of each solution in every PB is shown in Note S5 and Fig. S1 (ESI†), where it becomes clear that each PB is mainly originated by a handful of key environmental flows.

### Economic implications of planetary boundaries

**Fig. 5:**
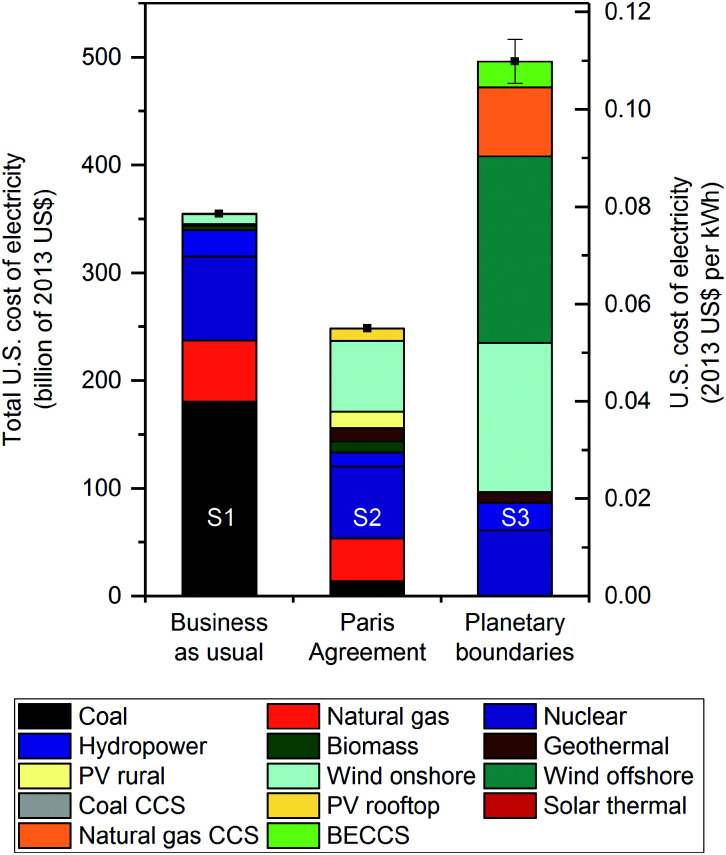
US optimal cost of electricity generation and imports in 2030 broken by technology. The primary *y*-axis denotes the total cost of electricity supply and the secondary *y*-axis represents the average cost of electricity in USD_2013_ per kWh. The business as usual energy mix, solution S1, (column on the left) represents an energy mix with the same technological breakdown as in 2012 but meeting the projected demand in 2030. The Paris Agreement mix, solution S2, (column on the center) represents the least cost energy mix in 2030 that would meet the Paris Agreement 2 degrees Celsius target. The planetary boundaries mix, solution S3, (column on the right) represents the least cost energy mix in 2030 that would minimize the transgression of planetary boundaries. Each error bar corresponds to one standard deviation due to the uncertainty in environmental burdens (*i.e.*, life cycle inventory entries) connected to planetary boundaries. The labeling of solutions is given in Table 2.

To understand the economic implications of meeting several PBs, we next compare the total US cost of electricity in solutions S1, S2 and S3 (Fig. 5). The Paris Agreement mix (S2) is less expensive than the BAU mix (S1), mainly due to the future economic competitiveness of the LCOE values of renewable technologies, particularly wind onshore and geothermal, in contrast to conventional power plants (Table S2, ESI†).^40^

The least cost solution that minimizes the transgression of PBs (S3) has the drawback of increasing the cost of electricity by 40% compared to the BAU mix (S1) while doubling the cost compared to the Paris Agreement mix (S2) (Fig. 5). The differences in costs incurred by solution S3 in contrast to the BAU (S1) and Paris Agreement (S2) mixes constitute 0.8% and 1.3%, respectively, of the US GDP in 2017. This extra cost is mainly due to the deployment of wind offshore, natural gas with CCS and BECCS, at present more expensive than other renewable technologies (*e.g.*, wind onshore and geothermal). The Paris Agreement mix is also cost optimal when no bounds on emissions are imposed, which highlights the economic^40^ (in addition to environmental) benefits of meeting the Paris Agreement 2 °C target.

Uncertainties associated with the future LCOE values, which are more pronounced in emerging technologies (*e.g.*, BECCS) than in conventional ones (Table S2, ESI†),^42^ are analyzed in Note S6 (ESI†). We note that emerging technologies are found to be necessary by ERCOM-PB to meet seven PBs concurrently (Fig. S2, ESI†); consequently, their uncertain LCOE values need to be properly assessed. Our analysis shows that these uncertainties associated with emerging technologies (*e.g.*, BECCS) deployed in solution S3 do not change substantially our economic results (Note S6 and Fig. S2, ESI†).

Furthermore, acknowledging that future LCOE values of emerging technologies might be critically affected by learning curves, we also performed a post-optimal uncertainty analysis of solutions S1 and S2 by varying the LCOE values of electricity technologies from the current levels^43^ to the future ones^40^ (Table S3, ESI†), where the latter values consider the expected potential technological development. Results reveal that the probability of the Paris Agreement mix (S2) being more expensive than the BAU mix (S1) is rather low (*i.e.*, 5%) (Note S7 and Fig. S3, ESI†). This is mainly due to the fact that electricity technologies performing very well in PBs (*e.g.*, wind onshore and geothermal) are also cost advantageous in the future compared to technologies dominating currently the BAU mix (Table S3, ESI†).

## Conclusions

Our results have strong implications for both policymaking and research on energy systems design. First, we found that the current US policy framework, solution S2, would ‘lock in’^44^ a plan that would not meet all PBs concurrently despite the attainment of the Paris Agreement 2 °C target; this finding reinforces the need to take action to keep our Earth within its ecological limits. Power plants are capital-intense investments, thus dismantling plants is seldom favorable. Therefore, designing early, comprehensive and strategic policies that could maintain our growing demand for energy without transgressing PBs is crucial for attaining sustainable development.

Wind onshore and offshore, natural gas with CCS and BECCS play a key role in meeting concurrently seven of the eight PBs considered in the US, while minimizing the transgression of the remaining PB on biogeochemical N flow. In fact, the ability to deploy negative emission technologies (*e.g.*, BECCS) in the power sector would provide some degree of flexibility in meeting the total atmospheric CO_2_ concentration and ocean acidification PBs at the global level, as the power sector could offset, to some extent, the contribution of other worse-endowed sectors to these PBs. Nonetheless, with the current technological landscape in the US power sector, meeting the global biogeochemical N flow seems unattainable; this is due to the limited potential of those technologies that are competitive across all PBs, such as hydropower and wind technologies. Pathways that could improve the current potential and reliability of hydropower and wind technologies through, for example, the deployment of more affordable storage facilities^45,46^ could aid meeting all of the PBs concurrently.

Alternatively, sectors (and also countries) could trade shares of PBs so one sector (or country) could operate under more stringent targets in those PBs easier to meet, while performing worse in those harder to satisfy. For example, the poor performance of energy systems in terms of biogeochemical N flow could be offset to some extent by imposing more stringent targets on other economic sectors with potential for offsetting such impact. Particularly, the power sector could exert a net negative contribution towards atmospheric CO_2_ concentration and ocean acidification by deploying BECCS, which would allow the sector to ‘trade’ shares of other PBs, mainly biogeochemical N flow, with other sectors. Nonetheless, this would ultimately require mechanisms to share burdens across sectors following some principles, which should consider the sectoral contributions to the population's well-being.

The reliance on expensive technologies (*e.g.*, wind offshore, natural gas with CCS and BECCS) needed to meet seven of the eight PBs while minimizing the transgression of the biogeochemical N flow PB would increase the cost of electricity generation, 40% relative to the BAU solution and 100% relative to the Paris Agreement solution. These additional costs relative to the BAU and Paris Agreement mixes would constitute 0.8% and 1.3% of the US GDP in 2017, respectively. Therefore, further research to decrease the LCOE of such technologies is needed to dampen the cost of meeting PBs. In this context, other negative emissions technologies, such as Direct Air Capture (DAC),^47^ could also be incorporated in ERCOM-PB. We keep the exploration of this idea as part of our future work due to the current limited availability of projected economic and technical data to the future of other negative emissions technologies.

We note that significant uncertainties are involved in the calculation of some PBs. For instance, the uncertainty range of the PB on the global P flow is a factor of ten, while the uncertainty range of the PB on ozone depletion is a factor of two.^18^ Narrowing the PB uncertainty ranges would have implications on the cost of generating sustainable electricity. For example, imposing less strict PBs on ESPs that are difficult to meet by the US power sector, such as climate change, could deploy less expensive electricity technologies and hence reduce the cost of electricity supply. Therefore, these uncertainties should be studied thoroughly to produce more robust planetary targets and to design sustainable energy systems and policies accordingly.

Finally, urgent research is needed to design characterization factors that link life cycle inventory entries to the PBs on the two ESPs overlooked so far, namely biosphere integrity and introduction of novel entities. Similarly, characterization models need to be designed to connect life cycle inventory entries readily available in LCA repositories (*e.g.*, ecoinvent^34,35^) to regional PBs. Lastly, global PBs on atmospheric aerosol loading and introduction of novel entities need to be quantified to sustain their safe operating space zones.

## Nomenclature

BAU: Business as usual
BECCS: Bio-energy with carbon capture and storage
CCS: Carbon capture and storage
DAC: Direct air capture
ERCOM: Emissions reduction cooperation model
ERCOM-PB: Emissions reduction cooperation model with planetary boundaries
ESM: Energy systems model
ESO: Electricity systems optimization
ESP: Earth-system process
GDP: Gross domestic product
GHG: Greenhouse gas
GVA: Gross value added
GWP100: 100-year global warming potential
INDC: Intended nationally determined contribution
LCA: Life cycle assessment
LCOE: Levelized cost of electricity
N: Nitrogen
P: Phosphorus
PB: Planetary boundary

### Indices

i
: Electricity generation technology
j
: State
p
: Planetary boundary

### Sets

Real numbers

### Parameters

*A*_*i*,*j*_
: Technical matrix of constraints for technology *i* in state *j*
*a*_*i*,*j*_
: Upper bound vector for technology *i* in state *j*
aS^USpower^: Assigned share of the full safe operating space to the power sector in the United States
*c* ^T^_*i,j*_
: Cost of electricity generation for technology *i* in state *j*
CF_*l*,*p*_: Characterization factor that links life cycle inventory entry *l* to planetary boundary *p*
EP_*i*,*j*,*p*_: Environmental burden linked to planetary boundary *p* per unit of energy supplied by technology *i* in state *j*
GVA^US^: United States total gross value added
GVA^USpower^: United States power sector gross value added
GWP100_*i*,*j*_: 100-year global warming potential per unit of energy generated by technology *i* in state *j*
LCI_*i*,*j*,*l*_: Life cycle inventory *l* generated when one unit of electricity is supplied *via* technology *i* in state *j*
POP^US^: United States population
POP^World^: World population
SOS_*p*_: Full safe operating space for planetary boundary *p*
SoSOS_*p*_: United States power sector absolute share of the full safe operating space for planetary boundary *p*
TARG: 100-year global warming potential target in line with the United States commitment to the Paris Agreement 2 degrees Celsius target in 2030

### Variables

COST^TOT^: Total cost of electricity supply
WT: Total weighted transgression of planetary boundaries by the United States power sector share of the safe operating space
PBT_*p*_: Transgression of downscaled planetary boundary *p*
*x*_*i*,*j*_
: Electricity generated by technology *i* in state *j*

## Conflicts of interest

There are no conflicts to declare.

## Supplementary Material

EE-012-C8EE03423K-s001
