# Supplementary material for: Powering sustainable development within planetary boundaries
Source: Energy Environ Sci. 2019 Jan 24;12(6):1890–900. doi: 10.1039/c8ee03423k (PMC6592157; doi:10.1039/c8ee03423k)
Supplement: EE-012-C8EE03423K-s001 [file EE-012-C8EE03423K-s001.pdf]

Electronic Supplementary Material (ESI) for Energy & Environmental Science.
This journal is © The Royal Society of Chemistry 2019

Powering sustainable development within planetary boundaries

Ibrahim M. Algunaibet,[a] Carlos Pozo,[a] Ángel Galán-Martín,[a] Mark A.J. Huijbregts,[b] Niall Mac Dowell [ac] and Gonzalo Guillén-Gosálbez *[ad]

[a.] Centre for Process Systems Engineering, Department of Chemical Engineering, Imperial College London, South Kensington Campus, London SW7 2AZ, UK

[b.] Department of Environmental Science, Institute for Water and Wetland Research, Radboud University, P.O. Box 9010, NL-6500, GL, Nijmegen, The Netherlands

[c.] Centre for Environmental Policy, Imperial College London, South Kensington Campus, London SW7 1NA, UK

[d.] Institute for Chemical and Bioengineering, Department of Chemistry and Applied Biosciences, ETH Zürich, Vladimir-Prelog-Weg 1, 8093 Zürich, Switzerland

* E-mail: g.guillen05@imperial.ac.uk

Supplementary Information

This document contains the supplementary material for the main manuscript. The document includes the following information. In Supplementary Note 1, we present the nomenclature of ERCOM-PB. In Supplementary Note 2, we present the mathematical formulation, methodology and data sources supporting ERCOM-PB. Supplementary Note 3 describes the uncertainty analysis. Supplementary Note 4 discusses the main limitations of our framework and the potential future work. Supplementary Note 5 highlights the environmental stressors causing the transgression of each planetary boundary in each solution. Supplementary Note 6 describes the economic uncertainty associated with meeting seven planetary boundaries concurrently. Supplementary Note 7 describes the uncertainty analysis results associated with the likelihood of the Paris Agreement solution being less expensive than the business as usual one.

Supplementary Note 1

Nomenclature

Indices

i	Power technologies
j	States in the United States
k	Southern Canadian regions
l	Life cycle inventory entries
p	Planetary boundaries

Sets

BT	Biomass and bio-energy with carbon capture and storage technologies
CT	coal with and without carbon capture and storage technologies
IR	Intermittent power technologies (i.e., photovoltaic, wind onshore and offshore, solar thermal and hydropower)
J	States considered (i.e., regions within the United States)
NGT	Natural gas with and without carbon capture and storage technologies
NC_j	Neighboring Canadian regions to state j
NU_j	Neighboring states to state j

Parameters

$aS^{USpower}$	Assigned share of the safe operating space to the power sector in the United States
BUC	Backup capacity share of dispatchable technologies that must be deployed for every non-dispatchable technology
$CAP_{i,j}^{CUR}$	Current installed capacity in 2012 of technology i in state j
$CAPF_{i,j}$	Capacity factor of technology i in state j
$CF_{l,p}$	Characterization factor that links life cycle inventory l to planetary boundary p
CO^{CAN}	Canadian import price of electricity
$CO_{i,j}^{CAP}$	Capital cost of technology i in state j
CO_{i}^{CAPAVE}	Average capital cost of technology i
$CO_{i,j}^{FIX}$	Fixed portion of the operating cost of technology i in state j
CO_{i}^{FIXAVE}	Average fixed portion of the operating cost of technology i
$CO_{i,j}^{VAR}$	Variable portion of the operating cost of technology i in state j
CO_{i}^{VARAVE}	Average variable portion of the operating cost of technology i
CTB	Maximum share of demand that can be met by electricity imports from Canadian regions
DEM_j	Electricity demand in state j
$DIST_{j,j'}$	Transmission distance between state j and state j'
$DISTCAN_{j,k}$	Transmission distance between state j and Canadian region k
DSF	Demand satisfaction factor
$EP_{i,j,p}$	Environmental burden linked to planetary boundary p per unit of electricity generated with technology i in state j

$EP_{i,k,p}$	Environmental burden linked to planetary boundary p per unit of electricity generated with technology i in Canadian region k
$GEN_{i,j}^{POT}$	Electricity generation potential with technology i in state j
GEN_{i}^{POTGLO}	National electricity generation potential with technology i in state j
$GWP100_{i,j}$	100-year global warming potential per unit of electricity generated with technology i in state j
GVA^{US}	Gross value added for the United States total economy in 2016
$GVA^{USpower}$	Gross value added for the United States power sector in 2016
H	Total number of hours in a given year, 8760 hours
$LCI_{i,j,l}$	Life cycle inventory entry l generated by one unit of electricity supplied with technology i in state j
$LCI_{i,k,l}$	Life cycle inventory entry l generated by one unit of electricity supplied with technology i in Canadian region k
SOS_p	Full safe operating space for every planetary boundary p
$SoSOS_p$	Absolute United States power sector share of the safe operating space for every planetary boundary p
$TARG$	United States pledged target governed by the Paris Agreement in 2030
TLF	Losses due to electricity generation, 0.62% for every 100 km
Pop^{US}	United States population in 2016
Pop^{World}	World population in 2016
ω_j	Cost adjustment factor corresponding to state j

Continuous variables

$CAP_{i,j}^{BU}$	Installed capacity of backup technology i in state j
$CAP_{i,j}^{ST}$	Installed capacity of standard technology i in state j
$COST_j$	Total cost of electricity generation in state j
$COST_j^{CAN}$	Cost of electricity imports from Canadian regions to state j
$COST_j^{CAP}$	Capital cost of electricity generation in state j
$COST_j^{FIX}$	Fixed cost of electricity generation in state j
$COST^{TOT}$	Total cost of electricity generation
$COST_j^{VAR}$	Variable cost of electricity generation in state j
EM_j	Total CO_2-Eq emissions of state j following the 100-year global warming potential
EP_p^{TOT}	Total Earth-system process performance linked to planetary boundary p
$GEN_{i,j}^{BU}$	Electricity generation by backup technology i in state j
$GEN_{i,j}^{ST}$	Electricity generation by standard technology i in state j
PBT_p	Transgression of planetary boundary p
$TRD_{j,j'}^{DEST}$	Net electricity imports from state j' to state j
$TRD_{j,j'}^{LOSS}$	Electricity transmission losses due to imports from state j' to state j
$TRD_{j',j}^{ORIG}$	Electricity exports from state j to state j'
$TRDCAN_{j,k}^{DEST}$	Electricity imports from Canadian region k to state j
$TRDCAN_{j,k}^{LOSS}$	Electricity transmission losses due to imports from Canadian region k to state j

$TRDCAN^{ORIG}_{j,k}$	Electricity exports from Canadian region k to state j
WT	Total weighted transgression of planetary boundaries by the United States power sector share of the safe operating space

Supplementary Note 2

Methodology and data sources: ERCOM-PB mathematical formulation

This section describes the modified mathematical formulation of the Emissions Reduction Cooperation Model (ERCOM)[1] that incorporates planetary boundaries, henceforth called ERCOM-PB. We also highlight the data sources used in the original ERCOM (Supplementary Table 1). The work by Galán-Martín et al.[1] offers further details about ERCOM's assumptions and data sources.

Here, we extend the original ERCOM[1] by incorporating additional equations and constraints that include planetary boundaries into the model. In this section, we describe ERCOM-PB, which contains blocks of equations characterizing environmental burdens, planetary boundaries, load-meeting constraints and equations required to assess the cost of electricity generation. ERCOM can be solved following a cooperative and non-cooperative mode between states[1], yet for simplicity, this contribution deals only with the full cooperative mode. Thereby, the results shown in this paper represent an upper bound on the economic and environmental benefits that could be realized. Only the equations and constraints related to the full cooperative mode are described here; the interested reader is referred to the work by Galán-Martín et al.[1] for further details on cooperation-related equations.

Greenhouse gas emissions constraints

The United States (US) has pledged to reduce its greenhouse gas emissions by 26-28% in 2025 compared to the 2005 levels using the 100-year Global Warming Potential (GWP100) (US Intended Nationally Determined Contribution (INDC) under the Paris Agreement 2 ˚C target[2]). Projecting this pledged mitigation effort linearly, as suggested by the published US INDC[2], results in a reduction target of 39% in 2030 compared to the 2005 levels. In this work, we assume that the reduction has to be met jointly by the US as a whole. States can, therefore, trade electricity or dispatchable resources to achieve the US Paris Agreement commitment. This constraint is only enforced in the Paris Agreement solution (S2) as follows:

$$\sum_j EM_j \leq TARG \qquad \text{(SE1)}$$

where EM_j is a continuous variable that represents the total CO_2-eq (as per the used GWP100 indicator) emitted by state j and $TARG$ is the US emissions reduction pledge governed by the Paris Agreement 2 ˚C target.

CO_2-eq emissions are calculated based on the total electricity generated by every technology in each state as follows:

$$EM_j = \sum_i \left(GEN_{i,j}^{ST} GWP100_{i,j}\right) + \sum_i \left(GEN_{i,j}^{BU} GWP100_{i,j}\right) \qquad \forall j \tag{SE2}$$

where $GEN_{i,j}^{ST}$ is the standard electricity generation with technology i in state j, $GWP100_{i,j}$ is the GWP100 environmental impact per unit of electricity generated with technology i in state j and $GEN_{i,j}^{BU}$ is the backup electricity generation with technology i in state j. Note that constraints (SE1) and (SE2) are used only when the Paris Agreement solution (S2) is computed.

Load meeting constraints

Electricity generation is limited by the generation potential available for each technology and state as follows:

$$GEN_{i,j}^{ST} + GEN_{i,j}^{BU} \leq GEN_{i,j}^{POT} \qquad \forall j, i \neq coal, coal\ CCS, natural\ gas, natural\ gas\ CCS, biomass, BE \tag{SE3}$$

where $GEN_{i,j}^{POT}$ is the electricity generation potential for technology i in state j. The constraint (SE3) is applicable for all technologies except for those that share the same energy feedstock, namely coal and coal with Carbon Capture and Storage (CCS), natural gas with and without CCS as well as biomass and Bio-energy with CCS (BECCS). These technologies are modeled as follows:

$$\sum_{i \in CT} \left(GEN_{i,j}^{ST} + GEN_{i,j}^{BU}\right) \leq GEN_{i',j}^{POT} \qquad \forall j, i' = coal \tag{SE4}$$

$$\sum_{i \in NGT} \left(GEN_{i,j}^{ST} + GEN_{i,j}^{BU}\right) \leq GEN_{i',j}^{POT} \qquad \forall j, i' = natural\ gas \tag{SE5}$$

$$\sum_{i \in BT} \left(GEN_{i,j}^{ST} + GEN_{i,j}^{BU}\right) \leq GEN_{i',j}^{POT} \qquad \forall j, i' = biomass \tag{SE6}$$

where the sets CT, NGT and BT represent coal-, natural gas- and biomass-powered technologies, respectively. Constraints (SE4), (SE5) and (SE6) ensure that technologies that share the same tradable energy feedstock do not exceed certain sensible limits on the generation potential in a given state. Such tradable energy resources are also bounded by their national availability, as stated in constraints (SE7-9).

$$\sum_{j}\sum_{i\in CT}\left(GEN_{i,j}^{ST}+GEN_{i,j}^{BU}\right)\leq GEN_{i'}^{POTGLO} \qquad \forall\, i'=coal \tag{SE7}$$

$$\sum_{j}\sum_{i\in NGT}\left(GEN_{i,j}^{ST}+GEN_{i,j}^{BU}\right)\leq GEN_{i'}^{POTGLO} \qquad \forall\, i'=natural\ gas \tag{SE8}$$

$$\sum_{j}\sum_{i\in BT}\left(GEN_{i,j}^{ST}+GEN_{i,j}^{BU}\right)\leq GEN_{i'}^{POTGLO} \qquad \forall\, i'=biomass \tag{SE9}$$

Installed capacity and electricity generation, both standard and backup, are linked as follows:

$$GEN_{i,j}^{ST}\leq CAP_{i,j}^{ST}CAPF_{i,j}H \qquad \forall i,j \tag{SE10}$$

$$GEN_{i,j}^{BU}=CAP_{i,j}^{BU}CAPF_{i,j}H \qquad \forall i,j \tag{SE11}$$

where $CAP_{i,j}^{ST}$ is a continuous variable that represents the standard installed capacity for technology i in state j, $CAP_{i,j}^{BU}$ is also a continuous variable that provides the backup installed capacity for technology i in state j, $CAPF_{i,j}$ denotes the capacity factor used to link the capacity to the generation with technology i in state j and H is a scalar that represents the total number of hours of operation in a year. The capacity factor represents the ratio between the actual and potential electricity output. This parameter limits the amount of electricity generated by each technology by considering those time periods in which the power plant is not operating. While the standard electricity generation is bounded by an inequality constraint (SE10), the backup electricity generation is modeled via an equality constraint (SE11) that ensures the grid reliability.

According to the US Energy Protection Agency, new nuclear power plants shall not be built[3]. This policy is modeled as follows:

$$CAP_{i,j}^{ST}+CAP_{i,j}^{BU}=CAP_{i,j}^{CUR} \qquad \forall j, i=\ nuclear \tag{SE12}$$

where $CAP_{i,j}^{CUR}$ is a parameter denoting the current installed nuclear capacity.

The integration of more intermittent energy resources into the grid inevitably decreases its reliability. Many studies dealt with this modeling issue. One way to model this is to assign each technology an inertia potential, which is lower for intermittent technologies than for those that are not intermittent[4]. Thereafter, the grid reliability is maintained by setting a global system inertia potential as a lower bound in the model, which should be met by the grid[4]. Similarly, we use the backup generation methodology to ensure that for every unit of electricity generated from a non-dispatchable resource, a certain proportion has to be generated with a dispatchable resource[5-7]. Both methodologies serve the same purpose, namely to maintain the reliability of the grid. Furthermore,

both methodologies indirectly increase the marginal cost of production, particularly if the dispatchable technology chosen to support the grid is more expensive than the non-dispatchable one. Dispatchable technologies include coal, coal with CCS, natural gas, natural gas with CCS, nuclear, biomass, BECCS and geothermal. On the other hand, non-dispatchable technologies include both rooftop and rural solar Photovoltaic (PV), both wind onshore and offshore, solar thermal and hydropower.

$$\sum_{i \notin IR} CAP^{BU}_{i,j} = BUC \sum_{i \in IR} CAP^{ST}_{i,j} \quad \forall j \tag{SE13}$$

The reliability of the grid is modeled using equation (SE13), where BUC is a scalar denoting the capacity share needed by dispatchable technologies for every unit of a non-dispatchable technology and IR is the set of non-dispatchable technologies. We also ensure that non-dispatchable technologies cannot generate backup electricity using equation (SE14).

$$CAP^{BU}_{i,j} = 0 \qquad \forall j, i \in IR \tag{SE14}$$

Electricity can only be traded between neighboring states and Canadian regions as follows:

$$TRD^{ORIG}_{j,j'} = 0 \qquad \forall j, j' \notin NU_j \tag{SE15}$$

$$TRDCAN^{ORIG}_{j,k} = 0 \qquad \forall j, k \notin NC_j \tag{SE16}$$

where $TRD^{ORIG}_{j,j'}$ represents electricity being imported by state j from state j', $TRDCAN^{ORIG}_{j,k}$ is the amount of electricity being imported by state j from the Canadian region k, NU_j is the set of neighboring states j' to state j and NC_j is the set of neighboring southern Canadian regions k to state j.

We also model the total electricity losses due to transmission as follows:

$$TRD^{ORIG}_{j,j'} = TRD^{DEST}_{j,j'} + TRD^{LOSS}_{j,j'} \qquad \forall j, j' \in NU_j \tag{SE17}$$

$$TRDCAN^{ORIG}_{j,k} = TRDCAN^{DEST}_{j,k} + TRDCAN^{LOSS}_{j,k} \qquad \forall j, k \in NC_j \tag{SE18}$$

where $TRD^{DEST}_{j,j'}$ is the total amount of electricity received at the final destination j from state j' and $TRD^{LOSS}_{j,j'}$ is the total amount of electricity losses due to electricity transmission from state j' to state j, $TRDCAN^{DEST}_{j,k}$ is the total amount of electricity received at the final destination j from the neighboring Canadian region k and $TRDCAN^{LOSS}_{j,k}$ is the total amount of electricity losses due to transmission from the neighboring Canadian region k to state j.

The total amount of electricity losses is modeled as a function of the distance and the amount of electricity being transmitted between regions as follows:

$$TRD^{LOSS}_{j,j'} = TRD^{ORIG}_{j,j'} DIST_{j,j'} TLF \qquad \forall j,j' \in NU_j \tag{SE19}$$

$$TRDCAN^{LOSS}_{j,k} = TRDCAN^{ORIG}_{j,k} DISTCAN_{j,k} TLF \qquad \forall j,k \in NC_j \tag{SE20}$$

where $DIST_{j,j'}$ is the distance between state j and neighboring state j', $DISTCAN_{j,k}$ is the distance between state j and neighboring Canadian region k and TLF is a scalar that links the electricity losses to the transmission distance.

To be consistent with the current energy market structure, total electricity imports from neighboring Canadian regions and states cannot exceed a given share of the total demand as follows:

$$\sum_{j,k \in NC_j} TRDCAN^{ORIG}_{j,k} \leq CTB \sum_j DEM_j \tag{SE21}$$

where CTB is a parameter that represents an upper bound on the total electricity demand that can be met by imports and DEM_j is the total electricity demand in state j.

To prevent states from acting as transmission nodes (i.e., import more electricity than the amount consumed to ultimately sell it), we constrain the total imports by each state by its total electricity demand (SE22).

$$\sum_{j' \in NU_j} TRD^{DEST}_{j,j'} + \sum_{k \in NC_j} TRDCAN^{DEST}_{j,k} \leq DEM_j \qquad \forall j \tag{SE22}$$

The domestic electricity generation plus the imports and minus the total exports must satisfy the total demand for that state adjusted by a reliability factor as follows:

$$\sum_i GEN^{ST}_{i,j} + \sum_{i \notin IR} GEN^{BU}_{i,j} + \sum_{j' \in NU_j} TRD^{DEST}_{j,j'} + \sum_{k \in NC_j} TRDCAN^{DEST}_{j,k} - \sum_{j' \in NU_j} TRD^{ORIG}_{j',j} = DEM_j DSF \tag{SE23}$$

where DSF is the demand satisfaction factor required to maintain the grid reliability forcing the system to deliver a reserve margin above the electricity demand.

Economic objective function and constraints

The objective function seeks to minimize the total cost of electricity generation as follows:

$$COST^{TOT} = \sum_j COST_j \tag{SE24}$$

where $COST^{TOT}$ is the total cost of electricity supply to the US and $COST_j$ is the cost of electricity generation in state j. The cost of electricity generation is structured as follows:

$$COST_j = COST^{CAP}_j + COST^{FIX}_j + COST^{VAR}_j + COST^{CAN}_j \quad \forall j \tag{SE25}$$

where $COST^{CAP}_j$ is the installed capacity cost in state j, $COST^{FIX}_j$ is the annual fixed cost in state j, $COST^{VAR}_j$ is the variable operating cost in state j and $COST^{CAN}_j$ is the electricity import cost from the neighboring Canadian regions to state j. The installed capacities of both standard and backup technologies determine the state capital cost as follows:

$$COST^{CAP}_j = \sum_i \left(\left(CAP^{ST}_{i,j} + CAP^{BU}_{i,j} \right) CO^{CAP}_{i,j} CAPF_{i,j} H \right) \quad \forall j \tag{SE26}$$

where $CO^{CAP}_{i,j}$ is a parameter that represents the unitary capital cost of technology i in state j. The fixed capacities of both standard and backup technologies determine the state annual fixed cost as follows:

$$COST^{FIX}_j = \sum_i \left(\left(CAP^{ST}_{i,j} + CAP^{BU}_{i,j} \right) CO^{FIX}_{i,j} CAPF_{i,j} H \right) \quad \forall j \tag{SE27}$$

where $CO^{FIX}_{i,j}$ is a parameter that represents the unitary annual fixed operating cost of technology i in state j. The variable costs of both standard and backup technologies determine the state variable operating cost as follows:

$$COST^{VAR}_j = \sum_i \left(\left(GEN^{ST}_{i,j} + GEN^{BU}_{i,j} \right) CO^{VAR}_{i,j} \right) \quad \forall j \tag{SE28}$$

where $CO^{VAR}_{i,j}$ is a parameter that represents the variable cost of technology i in state j. The total electricity imports from neighboring Canadian regions provide the total electricity import cost as follows:

$$COST^{CAN}_j = \sum_{k \in NC_j} TRDCAN^{DEST}_{j,k} CO^{CAN} \quad \forall j \tag{SE29}$$

where CO^{CAN} is a scalar that represents the Canadian unitary selling price of electricity.

Planetary boundaries constraints and objective function

Our work characterizes planetary boundaries and includes them into energy systems models. Each Earth-system process required to assess the performance of the grid in terms of planetary boundaries is modeled as follows:

$$EP^{TOT}_{p} = \sum_{i,j}\left(\left(GEN^{ST}_{i,j} + GEN^{BU}_{i,j}\right)EP_{i,j,p}\right) + \sum_{j,k}\left(TRDCAN^{ORIG}_{j,k}EP_{i',k,p}\right) \qquad \forall p,i' = hydropower \tag{SE30}$$

where EP^{TOT}_{p} represents the total performance of each Earth-system process linked to planetary boundary p and $EP_{i,j,p}$ is a parameter denoting the total environmental burden linked to planetary boundary p per unit of energy generated with technology i in state j and $EP_{i',k,p}$ is a parameter denoting the total environmental burden linked to planetary boundary p of technology i' in Canadian region k. Note electricity imports from neighboring Canadian regions are assumed to be generated from hydropower plants[1] as shown in Equation (SE30). The environmental burden must be determined from the life cycle inventory entries associated with each technology and region, which provide the life cycle emissions to air, soil and water per unit of electricity generated by that specific technology as follows:

$$EP_{i,j,p} = \sum_{l}\left(CF_{l,p} \cdot LCI_{i,j,l}\right) \qquad \forall p,i,j \tag{SE31}$$

$$EP_{i,k,p} = \sum_{l}\left(CF_{l,p} \cdot LCI_{i',k,l}\right) \qquad \forall p,k,i' = hydropower \tag{SE32}$$

where $CF_{l,p}$ is the characterization factor that links life cycle inventory entry l to planetary boundary p, $LCI_{i,j,l}$ is the life cycle inventory entry l generated by one unit of electricity supplied with technology i in state j and $LCI_{i',k,l}$ is the life cycle inventory entry l generated by one unit of electricity supplied with technology i' in Canadian region k.

The US power sector share of the safe operating space linked to each planetary boundary connected to the Earth-system processes considered in this work are obtained as follows:

$$aS^{USpower} = \frac{Pop^{US}}{Pop^{World}} \cdot \frac{GVA^{USpower}}{GVA^{US}} \tag{SE33}$$

$$SoSOS_p = \mathrm{aS}^{USpower} \cdot \mathrm{SOS}_p \qquad \forall p \tag{SE34}$$

$$EP^{TOT}_{p} \leq SoSOS_p + PBT_p \qquad \forall p \tag{SE35}$$

$$PBT_p \geq 0 \qquad \forall p \tag{SE36}$$

where $aS^{USpower}$ is the assigned share of the safe operating space to the US power sector, Pop^{US} is the US population in 2016, Pop^{World} is the world population in 2016, $GVA^{USpower}$ is the gross value added

for the US power sector in 2016, GVA^{US} is the gross value added for the US total economy in 2016, $SoSOS_p$ is the US power sector absolute share of the safe operating space for every planetary boundary p, SOS_p is the full safe operating space for every planetary boundary p and PBT_p is a positive variable that quantifies the transgression of planetary boundary p.

The total weighted transgression of every planetary boundary by its corresponding US power sector share of the safe operating space, which is minimized in the planetary boundaries solution (S3) and only quantified in the other solutions (i.e., not optimized), is obtained as follows:

$$WT = \sum_{p} \left(\frac{PBT_p}{SoSOS_p} \right) \tag{SE37}$$

where WT is the summation of the normalized transgression of each planetary boundary p by its corresponding US power sector share of the safe operating space. Equation (SE37) is, therefore, the objective function of the minimization problem in the planetary boundaries solution (S3).

The following models are solved in the main manuscript to obtain three solutions namely S1, S2 and S3:

- To compute solution S1, which corresponds to the US 2012 default developments in the power sector to meet the 2030 electricity demand, the share of each technology is fixed to its 2012 level and the demand is projected to 2030.
- To compute solution S2, which represents the least cost solution that meets the US commitment to the Paris Agreement, we minimize equation SE24, subject to constraints (SE1-SE29).
- To compute solution S3, which corresponds to the system minimizing the transgression of planetary boundaries at minimum cost, we minimize equation SE37, subject to constraints (SE3-SE37). We then take the value of the total normalized transgression, i.e., WT in equation (SE37), and define this value as an upper bound (i.e., it should never be exceeded by the optimal solution of the model) in another model that minimizes equation S24 subject to constraints (SE3-SE37).

ERCOM-PB is modeled in the General Algebraic Modeling System (GAMS)[8] version 25.0.2. It features 12,802 continuous variables and 8,941 constraints. The GUROBI solver version 7.5.2 was used to solve the linear programming model on an Intel®Core™ i7-6700 processor operating at 3.40 GHz, taking less than one CPU second in the different instances.

Supplementary Note 3

Uncertainty analysis approach

We perform three cases of uncertainty analyses:

- The first case deals with the uncertainties associated with life cycle inventory entries linked to planetary boundaries. Here, we assume that life cycle inventories follow a lognormal distribution and we solve the optimization model at every iteration. The results are shown in the figures in the main manuscript where each error bar represents one standard deviation.
- The second case deals with the impact of the uncertainties associated with the future Levelized Cost of Electricity (LCOE) values on meeting seven planetary boundaries concurrently. Here, we assume that LCOE values follow a uniform distribution where the ranges are reported in Supplementary Table 2 and solve the optimization model at every iteration. The results are shown in Supplementary Figure 2 and discussed in Supplementary Note 6.
- The third case deals with the impact of the uncertainties associated with the LCOE values due to the consideration of learning curves. To be more precise, we aim to quantify the likelihood of the Paris Agreement mix, solution (S2), being less expensive than the Business as Usual (BAU) mix, solution (S1). Here, we assume that LCOE values follow a uniform distribution where the ranges are tabulated in Supplementary Table 3 and perform a post-optimal analysis (i.e., optimal mixes are fixed to solutions S1 and S2 and then the LCOE values are varied between the current to future levels). The results are shown in Supplementary Figure 3 and discussed in Supplementary Note 7.

Uncertainty analysis approach corresponding to the life cycle inventory entries (results shown in the main body of the manuscript)

To assess the impact of the uncertainties involved in the life cycle inventory entries used to evaluate the performance of Earth-system processes on planetary boundaries, we generated 100 scenarios and solved the optimization model (i.e., ERCOM-PB) for each of them separately. Each scenario corresponds to a different materialization of all the uncertain parameters, i.e., life cycle inventory entries, simultaneously generated by applying Monte Carlo sampling on the probability distributions of the inventory parameters. This number of scenarios meets the Law and Kelton's test[9], that is, it satisfies a confidence level of 95% with a relative error below 5% defined on the optimal objective function value. In Figures 2, 3, 4 and 5 in the manuscript, we show the results of the uncertainty analysis as error bars, where each error bar represents one standard deviation. We note that we apply the uncertainty analysis to the three solutions analyzed in the work (i.e., S1, S2 and S3).

Following standard practices in the life cycle assessment literature, each life cycle inventory entry (i.e., life cycle emissions to air, soil and water associated with each technology and required to

evaluate their performance in terms of planetary boundaries) was assumed to follow a lognormal cumulative distribution function[10]. That is, the natural logarithm of the life cycle inventory entry l associated with technology i in state j follows a normal distribution with mean $\mu_{l,i,j}$ and standard deviation $\sigma_{l,i,j}$. The geometric distribution $\sigma^{geo}_{l,i,j}$ of each uncertain life cycle inventory entry is obtained as follows:

$$\sigma^{geo}_{l,i,j} = \sqrt{e^{\sqrt{\begin{matrix}[\ln(U_{1,l,i,j})]^2 + [\ln(U_{2,l,i,j})]^2 + [\ln(U_{3,l,i,j})]^2 + [\ln(U_{4,l,i,j})]^2 + \\ [\ln(U_{5,l,i,j})]^2 + [\ln(U_{6,l,i,j})]^2 + [\ln(U_{b,l,i,j})]^2\end{matrix}}}} \qquad \forall l,i,j \tag{SE38}$$

where $\sigma^{geo}_{l,i,j}$ is the geometric standard deviation of the lognormal cumulative distribution function for each life cycle inventory entry l of technology i in state *j*, $U_{1,l,i,j}$ to $U_{6,l,i,j}$ are the scores used in the Pedigree matrix for each life cycle inventory entry l of technology i in state *j* and $U_{b,l,i,j}$ is the basic uncertainty factor used in the Pedigree matrix for each life cycle inventory entry l of technology i in state *j*. Further details on all the U values can be found in the SimaPro manual[11].

The standard deviation of the uncertain parameter's natural logarithm is obtained as follows:

$$\sigma_{l,i,j} = \ln\left(\sigma^{geo}_{l,i}\right) \qquad \forall l,i,j \tag{SE39}$$

while the mean of the uncertain parameter's natural logarithm $\mu_{l,i,j}$ is computed from its expected value and the variable's natural logarithm standard deviation as follows:

$$\mu_{l,i,j} = \ln\left(E[LCI_{l,i,j}]\right) - \frac{(\sigma_{l,i,j})^2}{2} \qquad \forall l,i,j \tag{SE40}$$

where $E[LCI_{l,i,j}]$ is the expected value of the uncertain parameter (i.e., arithmetic mean), retrieved from life cycle assessment repositories for each life cycle inventory l, technology i in state j. We finally derive the random values of the life cycle inventory entries by sampling on the lognormal distribution with parameters $\mu_{l,i,j}$ and $\sigma_{l,i,j}$ ($LCI_{l,i,j} \sim lognormal(\mu_{l,i,j}, {\sigma_{l,i,j}}^2)$). Note that we assume independent uncertain parameters, so a different seed is applied to sample each life cycle inventory entry separately. We applied the same approach to calculate the random life cycle inventory entries associated with electricity generation in neighboring Canadian regions.

Uncertainty analysis approach corresponding to the levelized cost of electricity (results shown only in this supplementary information and discussed in detail in Supplementary Notes 6 and 7)

We use two sets of LCOE values to (i) asses the uncertainty associated with future LCOE on meeting seven planetary boundaries concurrently, i.e., solution S3, (Supplementary Table 2) and (ii) quantify

the impact of learning curves associated with the LCOE on the likelihood of the Paris Agreement, solution (S2), being more expensive than the BAU, solution (S1), (Supplementary Table 3). To this end, we also ran ERCOM-PB 100 times, where each run entails specific values of the LCOE of the different technologies within the bounds reported in Supplementary Table 2 (analysis (i)). On the other hand, we performed a post-optimal analysis in analysis (ii) where we fix the mixes corresponding to solutions S1 and S2 and then vary the LCOE values reported in Supplementary Table 3. The number of scenarios was defined to meet the Law and Kelton's test[9] with a confidence level of 95% and a relative error below 5%. The results of analysis (i) are reported in Supplementary Figure 2 and discussed in Supplementary Note 6. The results of analysis (ii) are reported in Supplementary Figure 3 and discussed in Supplementary Note 7.

We assume that the LCOE follows a uniform distribution. To generate the Monte Carlo samples following a uniform distribution, two parameters are needed, namely a lower and upper bound for every electricity technology (Supplementary Table 2 and Supplementary Table 3). The probability density function for the uniform distribution can be defined as follows:

$$f(x) = \begin{cases} \dfrac{1}{(b-a)} & a \leq x < b \\ 0 & otherwise \end{cases} \qquad \text{(SE41)}$$

where x is the random value, a is the lower bound (minimum LCOE value) and b is the upper bound (maximum LCOE value).

Supplementary Table 2 and Supplementary Table 3 report the bounds for the total LCOE values. Nonetheless, each LCOE value could be divided into (i) capital and transmission lines, (ii) fixed operating and maintenance and (iii) variable operating and maintenance costs. Following Galán-Martín et al.[1], we assume the portion of each of these costs stay fixed to the average values reported by the US Energy Information Administration (EIA)[12]. Consequently, at each run, we vary the total LCOE values and then calculate each cost type. Each cost component is needed in ERCOM-PB as the fixed costs are linked to the plant capacity and the variable ones are linked to electricity generation.

The LCOE value differs from one state to another due to, for example, the disparity in the endowment of energy sources and labor markets between states. Following Galán-Martín et al.[1], we regionalize each random LCOE value to each state as follows:

$$CO_{i,j}^{CAP} = CO_{i}^{CAPAVE} \omega_j \left[\frac{|J|}{\sum_{j'} 1/CAPF_{i,j'}} \frac{1}{CAPF_{i,j}} \right] \qquad \forall i,j \qquad \text{(SE42)}$$

$$CO_{i,j}^{FIX} = CO_{i}^{FIXAVE} \omega_j \left[\frac{|J|}{\sum_{j'} 1 / CAPF_{i,j'}} \frac{1}{CAPF_{i,j}} \right] \qquad \forall i,j \qquad \text{(SE43)}$$

$$CO_{i,j}^{VAR} = CO_{i}^{VARAVE} \omega_j \left[\frac{|J|}{\sum_{j'} 1 / CAPF_{i,j'}} \frac{1}{CAPF_{i,j}} \right] \qquad \forall i,j \qquad \text{(SE44)}$$

where CO_{i}^{CAPAVE}, CO_{i}^{FIXAVE} and CO_{i}^{VARAVE} are the average capital, fixed operating and maintenance and variable operating and maintenance costs, respectively, per unit of electricity generated with technology i, ω_j is the cost adjustment factor corresponding to state j and $|J|$ is the cardinal (i.e., size) of set J that represents the states considered in the model.

Supplementary Note 4

Limitations and future work

The following limitations and assumptions of our work are acknowledged:

- We acknowledge that our allocation methodology assumes static shares of planetary boundaries. Nonetheless, those shares could be dynamic in nature based on the 'elasticity'[13] of each sector, that is, on its ability to reduce its burdens while maintaining a given level of outputs. For example, the power sector has less technological resilience towards reducing the biogeochemical Nitrogen (N) flow, since most of these environmental burdens are generated earlier in the supply chain. Meanwhile in the power sector, it might be easier to find more technological options to decrease its CO_2 emissions, this sector could ultimately trade part of its planetary boundaries on climate change with the agricultural sector, if the latter shows technological resiliency to meet a stricter planetary boundary on biogeochemical N flow. We keep this sectoral dynamic share allocation process as part of the future work since the main objective of this paper is to motivate the incorporation of planetary boundaries into energy systems models.
- Data for applying the sharing principle chosen in this work (i.e., population and gross value added) should, in principle, correspond to year 2030 to reflect upon the time duration of the analysis. However, forecasts for sectoral gross value added for 2030 are at present unavailable. Hence, the latest data for population and gross value added (i.e., 2016) were used instead in the study. Moreover, population and gross value added vary over time and, therefore, the shares derived from such indicator should be updated periodically.
- According to the PB-LCIA methodology[14], freshwater withdrawal (widely available in life cycle repositories, such as ecoinvent[10, 15]) is used to characterize the environmental flows related to the

planetary boundary on global freshwater use. Nonetheless, there could be a slight mismatch between freshwater consumption and freshwater withdrawal, since part of the withdrawn freshwater could potentially be recycled[16]. Unfortunately, freshwater consumption data for each electricity technology are currently unavailable.

- Regional planetary boundaries were omitted from the study due to the lack of applicable life cycle inventory data in life cycle repositories. In addition, characterization models to quantify the change in biosphere integrity are currently unavailable; consequently, we omitted the two planetary boundaries on biosphere integrity from the analysis[14]. Furthermore, there is no global planetary boundary on atmospheric aerosol loading, although characterization factors have been defined for a set of substances; hence, it was also omitted in our analysis[17]. Lastly, due to the lack of a control variable and planetary boundary on the introduction of novel entities, we discarded this planetary boundary in the analysis[17]. Our method would be nevertheless able to deal with these planetary boundaries, once the aforementioned planetary boundaries, their associated characterization models and applicable life cycle inventory data become available.

Supplementary Note 5

Environmental flows contribution to the transgression of planetary boundaries

Supplementary Figure 1 shows the performance of each environmental flow linked to every planetary boundary for each mix relative to its corresponding downscaled planetary boundary applicable to the US power sector. CO_2 dominates the environmental flows that cause the transgression of both climate change planetary boundaries and the planetary boundary on ocean acidification in both the BAU mix (solution S1) and the Paris Agreement mix (solution S2). In the planetary boundaries mix (solution S3), CO_2 shows a negative contribution due to the deployment of BECCS, whereas CH_4 dominates the positive impact on climate change and ocean acidification.

Across all solutions, NO_x and NO_3^- dominate the transgression of the planetary boundary on global biogeochemical N flow. Freshwater causes the transgression of the planetary boundary on global freshwater consumption in the BAU mix (S1) as well as in the Paris Agreement mix (S2), since it is the only environmental flow connected to the planetary boundary on freshwater consumption. Stratospheric ozone depletion is transgressed only in the BAU solution (S1) due mainly to N_2O emissions to air, which were not considered in the method we followed to translate life cycle inventory entries into planetary boundaries[14]. Therefore, we recommend the consideration of the N_2O emissions impact on the ozone layer due to their noticeable contribution found in our work. Planetary boundaries on global biogeochemical Phosphorus (P) flow and land-system change are not transgressed across all the considered energy mixes, which reflects upon the low quantities of

environmental flows linked to electricity technologies that are connected to such respected planetary boundaries.

Supplementary Note 6

Implications on the cost of meeting planetary boundaries considering uncertainties in the future LCOE values (analysis (i) in Supplementary Note 3)

Since the analysis is performed in year 2030, technological improvements that would make the price of emerging electricity technologies more competitive could materialize. In this supplementary note, therefore, we analyze the uncertainties associated with the future LCOE values of all electricity technologies simultaneously (analysis (i)). More precisely, we are interested in evaluating the extent to which the uncertainties associated with the future LCOE values of all electricity technologies could influence the cost of meeting seven planetary boundaries concurrently. We do so by varying the LCOE values between the ranges reported in Supplementary Table 2 following a uniform distribution where we solve the optimization model at every iteration.

Supplementary Figure 2 shows the results of the uncertainty analysis of the total cost of electricity and the performance on each planetary boundary due to the uncertainty associated with the LCOE values of electricity technologies. In other words, the results describe what happens in terms of the total cost of electricity and performance on every planetary boundary of the solutions found when the LCOE values are changed in the model as a result of different materializations of the associated uncertainty (Supplementary Table 2).

Our main results clearly show that BECCS power plants would play a critical role in meeting climate change planetary boundaries in the future; this is due to their negative life cycle CO_2 footprint when the whole supply chain is considered. However, the future LCOE of BECCS remains uncertain[18], particularly, when technological improvements are considered (Supplementary Table 2). Here, our uncertainty analysis shows that within the limits defined for the LCOE of BECCS and other electricity technologies, ERCOM-PB will always deploy BECCS in the planetary boundaries mix (S3) (Supplementary Figure 2). This is because BECCS is required to meet the CO_2-driven planetary boundaries, defined on climate change and ocean acidification, which cannot be met with other technologies. This insight should encourage investors to explore pathways to decrease the cost of BECCS to sustain the Earth's ecological capacity without significantly exacerbating our economic welfare. Overall, one standard deviation corresponds to 5% of the mean of the cost of meeting seven planetary boundaries concurrently, i.e., solution (S3), (Supplementary Figure 2). Therefore, our economic insights are robust within the assumptions highlighted in Supplementary Table 2.

Supplementary Note 7

Likelihood of the Paris Agreement mix being more expensive than the business as usual mix: post-optimal analysis focusing on learning curves (analysis (ii) in Supplementary Note 3)

When future energy mixes are studied, their future LCOE values should be considered to derive sound insights. Typically, learning curves are used to quantify the LCOE reductions for renewable technologies that currently might not be competitive when compared to conventional ones. Many studies concluded that the future LCOE of some renewable technologies (e.g., wind onshore and geothermal) would be more competitive than those of conventional ones (e.g., coal power plants)[12, 19].

Here, we are interested in evaluating the extent to which the Paris Agreement mix (solution S2) is better in economic terms than the BAU mix (solution S1). To this end, the outcome of learning curves is varied by performing a post-optimal analysis on the mixes found. First, we fix the mixes found with the average LCOE values (third column in Supplementary Table 2). Thereafter, we vary the LCOE values in the ranges reported in Supplementary Table 3 following a uniform distribution where the maximum value corresponds to the current LCOE values[20] and the minimum value corresponds to the expected LCOE values in the future in line with learning curve studies[12]. The rationale for this is to consider that, departing from the current cost, the technologies could evolve in different ways to finally reach an LCOE in between the cost today and the projected cost considering learning curves in the targeted year. Finally, for every scenario we divide the cost of the Paris Agreement solution (S2) by that of the BAU solution (S1) and show the results in Supplementary Figure 3, where a value greater than one indicates that the Paris Agreement solution is more expensive than the BAU solution, while a value less than one indicates the converse.

The Paris Agreement mix (S2) is found to be less costly than the BAU mix (S1) in 95 out of the 100 scenarios (i.e., runs) considered (Supplementary Figure 3). This is due to the competitiveness of some electricity technologies in the future, primarily wind onshore and geothermal – both combined representing only 4% of the BAU mix compared to 32% in the Paris Agreement solution. Therefore, despite the uncertainty associated with the LCOE of renewable technologies in the future, the Paris Agreement mix remains less expensive than the BAU mix with a probability of 95%.

The economic argument of keeping existing coal power plants in the 2012 mix focuses on the economic advantage of some coal plants that are fully depreciated and still under operation. For instance, the LCOE of a fully depreciated coal power plant is around half of a new one[19]. LCOE values are designed considering a 30-year cost recovery period[12]; hence, when plants operate beyond such

period their LCOE values drop giving them an economic advantage over renewables that are just starting to enter the market. In 2030, however, most of these old fully depreciated conventional power plants would be retired and hence economic renewables would constitute a greater share of the energy mix.

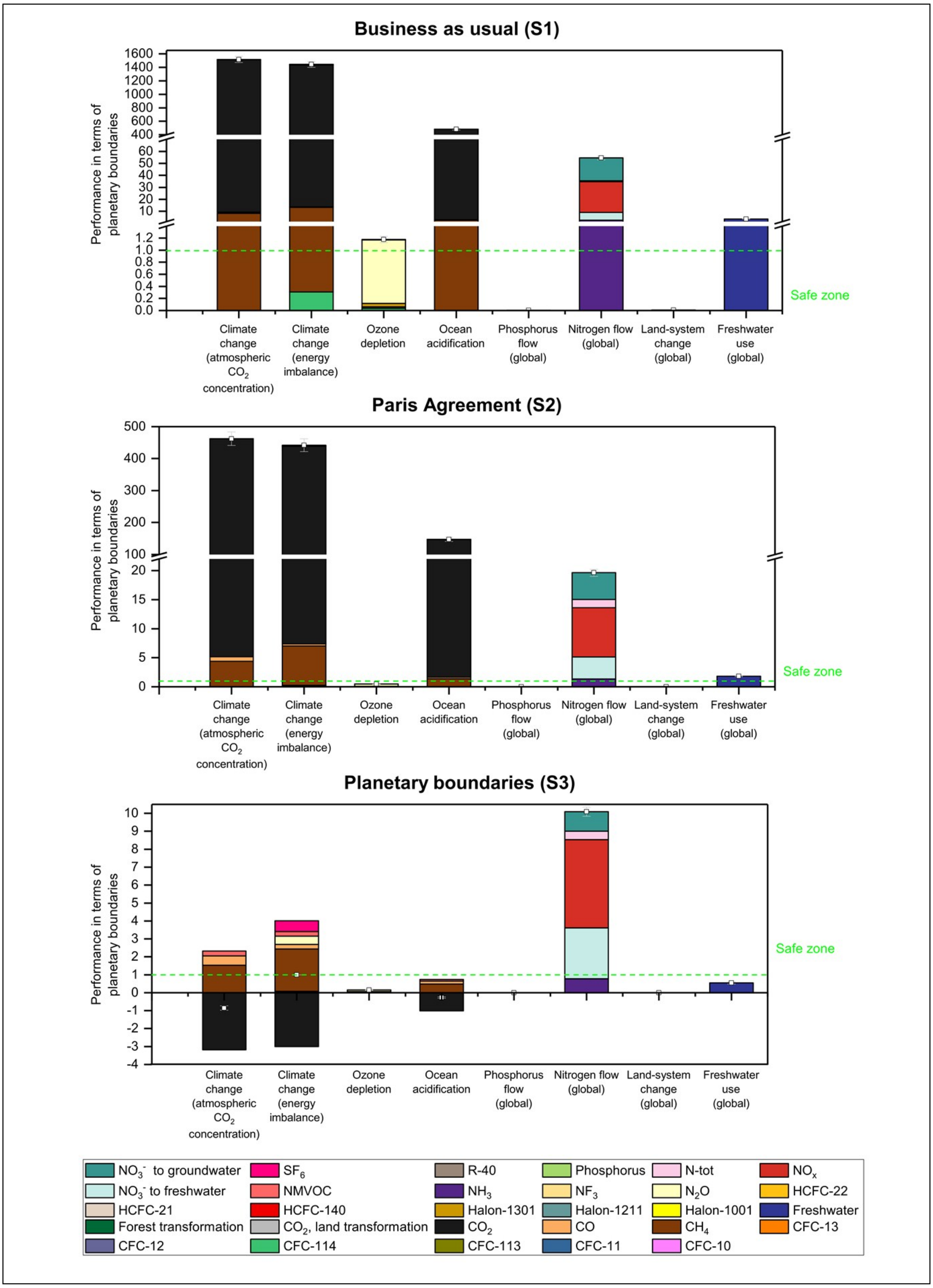

Supplementary Figure 1 Global US electricity generation and imports performance relative to downscaled planetary boundaries broken down by environmental flows. The ‘Business as usual’ energy mix, solution S1, (bars on the top) represents the 2012 energy mix in 2030. The ‘Paris Agreement’ mix, solution S2, (bars on the center) represents the least cost energy mix in 2030 that would meet the Paris Agreement. The ‘Planetary boundaries’ mix, solution S3, (bars on the bottom) represents the least cost energy mix in 2030 that would minimize the transgression of planetary boundaries.

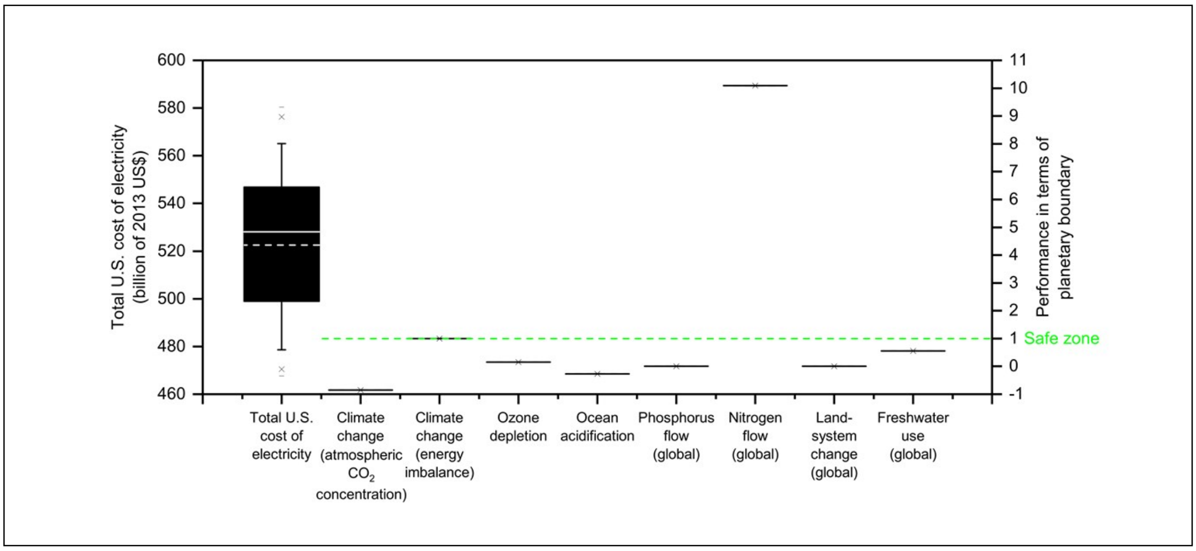

Supplementary Figure 2 Box plot with whisker summarizing the uncertainty analysis of the ‘Planetary boundaries’ mix, solution S3, which represents the least cost energy mix in 2030 that would minimize the transgression of planetary boundaries, due to the uncertainty in the future levelized cost of electricity values (Supplementary Table 2). Whiskers represent the range from 5% to 95%. The dashed white line represents the mean and the solid white line represents the median. The ‘x’ sign represents the last 1% of the results and the ‘-’ sign represents the maximum and minimum values in the sample. The x-axis shows the implications of the uncertainty in the future levelized cost of electricity values on the cost of US electricity generation and imports (primary y-axis), as well as on the performance relative to downscaled planetary boundaries (secondary y-axis).

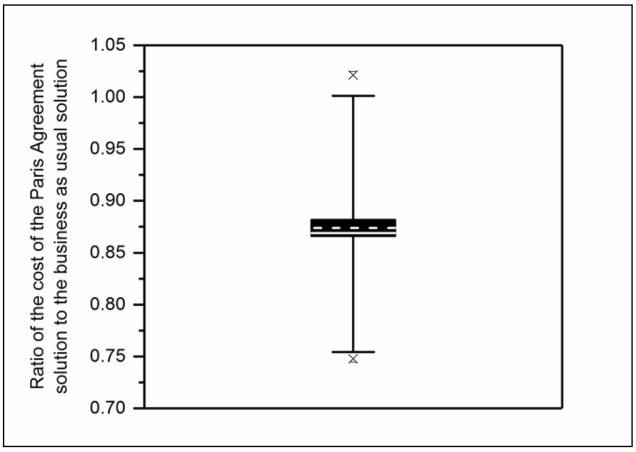

Supplementary Figure 3 Box plot with whisker summarizing the likelihood of the Paris Agreement solution (S2) being more expensive than the business as usual solution (S1) due to the uncertainty in the levelized cost of electricity induced by the consideration of learning curves (Supplementary Table 3). Whiskers represent the range from 5% to 95%. The dashed white line represents the mean and the solid white line represents the median. The ‘x’ sign represents the last 1% of the results and the ‘-’ sign represents the maximum and minimum values in the sample.

Supplementary Table 1 Data sources for the parameters used in ERCOM-PB, more details are available in the work by Galán-Martín et al.[1]

Parameter	Source	Description
BUC	Brown et al.[7]	For every unit of capacity from a non-dispatchable technology, 50% has to be built by dispatchable technologies to maintain the grid reliability
$CAP_{i,j}^{CUR}$	EIA[21]	Current United States installed capacity in 2012
$CAPF_{i,j}$	EIA[21]	Capacity factor
$CF_{l,p}$	Ryberg et al.[14]	Characterization factor that links life cycle inventory entry to planetary boundary
CO^{CAN}	CEA[22]	Canadian import price of electricity, 39 USD_{2013}/MWh
$CO_{i,j}^{CAP}$	BECCS: Cuellar and	Capital cost
$CO_{i,j}^{FIX}$	Herzog[23] and EIA[12]	Fixed portion of the operating cost
$CO_{i,j}^{VAR}$	Others: EIA[12]	Variable portion of the operating cost
CTB	NERC[24]	Maximum amount of electricity imports from Canadian regions, 5%
DEM_j	EIA[21]	Electricity demand
$DIST_{j,j'}$	Galán-Martín et al.[1]	Total transmission distance between states
$DISTCAN_{j,k}$		Total transmission distance between states and Canadian regions
DSF	Short et al.[25]	Demand satisfaction factor, 1.05
$GEN_{i,j}^{POT}$	NERL[26]	Potential of electricity generation
GEN_{i}^{POTGLO}		Total United States potential of electricity generation
GVA^{US}	United Nations[27]	Gross value added for the United States total economy in 2016
$GVA^{USpower}$	United Nations[27]	Gross value added for the United States power sector in 2016
$GWP100_{ij}$	*Life cycle data:* Coal with CCS:	100-year global warming potential per unit of electricity generated
$LCI_{i,j,l}$	Iribarren et al.[28]	Life cycle inventory entry per unit of electricity generated
$LCI_{i,k,l}$		
	Solar thermal: Corona et al.[29]	
	Natural gas with CCS: Petrakopoulou et al.[30]	
	BECCS: Oreggioni et al.[31]	
	Remaining technologies: ecoinvent[10, 15]	
Pop^{US}	World Bank Group[32]	United States population in 2016
Pop^{World}	World Bank Group[32]	World population in 2016
SOS_p	Steffen et al.[17] and Ryberg et al.[33]	Full safe operating space for every planetary boundary
$TARG$	Paris Agreement[2]	Paris Agreement target in 2030
TLF	Fripp[34]	Losses due to electricity generation, 0.62% for every 100 km
ω_j	U.S. Army Corps of Engineers[35]	Cost adjustment factor for every state

Supplementary Table 2 Future average levelized cost of electricity for each technology and uncertainty ranges.

[a] We use the share of the additional cost caused by adding a CCS unit to a biomass power plant for every cost component reported by Cuellar and Herzog[23] and multiply the shares by the biomass power plant cost components reported by EIA[12]. We assume 19% reduction from the resultant current levelized cost of electricity of BECCS to the one in 2030 in line with EIA projections[12, 20] for biomass plants.

[b] We assume 30% of the cost corresponding to the capital cost of the CCS unit to follow the range reported by Koelbl et al.[18] (±25% of the mean) and the remaining 70% of the cost to follow the range reported by EIA[12] for biomass power plants.

Technology	Lower bound on the future total levelized cost of electricity (USD_{2013}/MWh)	Average future total levelized cost of electricity (USD_{2013}/MWh)	Upper bound on the future total levelized cost of electricity (USD_{2013}/MWh)	Source
Coal	87.1	95.2	119	EIA[12]
Natural gas	70.4	75.1	85.5	EIA[12]
Nuclear	91.8	95.2	101	EIA[12]
Hydropower	69.3	83.6	107.2	EIA[12]
Biomass	90	100.4	117.4	EIA[12]
Geothermal	43.8	47.8	52.1	EIA[12]
PV rural	97.8	125.3	193.3	EIA[12]
Wind onshore	65.6	73.6	81.6	EIA[12]
Wind offshore	169.5	196.9	269.8	EIA[12]
Coal CCS	132.9	144.4	160.4	EIA[12]
PV rooftop	97.8	125.3	193.3	EIA[12]
Solar thermal	174.4	239.7	382.5	EIA[12]
Natural gas CCS	93.3	100.2	110.8	EIA[12]
BECCS*	191.7[b]	224.3[a]	267.3[b]	Average value: Cuellar and Herzog[23] and EIA[12]<br>Bounds: Koelbl et al.[18] and EIA[12]

Supplementary Table 3 Levelized cost of electricity without (current) and with (future) the consideration of potential technological improvements (i.e., learning curves). Only levelized cost of electricity values for technologies deployed in mixes S1 and S2 are reported.

Technology	Current levelized cost of electricity[20], no learning curves (USD_{2013}/MWh)	Future levelized cost of electricity[12], with learning curves (USD_{2013}/MWh)
Coal	108.6	95.2
Natural gas	89.9	75.1
Nuclear	128.8	95.2
Hydropower	129.7	83.6
Biomass	120.1	100.4
Geothermal	125.2	47.8
PV rural	428.6	125.3
Wind onshore	161.5	73.6
PV rooftop	428.6	125.3
Solar thermal	277.6	239.7

Supplementary References

1. A. Galan-Martin, C. Pozo, A. Azapagic, I. E. Grossmann, N. Mac Dowell and G. Guillen-Gosalbez, *Energy & Environmental Science*, 2018, **11**, 572-581.

2. UNFCCC, *United States intended nationally determined contribution under the Paris Agreement*, United Nations Framework Convention on Climate Change, 2015. https://www4.unfccc.int/sites/ndcstaging/PublishedDocuments/United%20States%20of%20America%20First/U.S.A.%20First%20NDC%20Submission.pdf.

3. EPA, *Clean Power Plan for existing Power Plants. Environmental Protection Agency*, 2015. https://archive.epa.gov/epa/cleanpowerplan/clean-power-plan-existing-power-plants-regulatory-actions.html.

4. C. F. Heuberger, I. Staffell, N. Shah and N. Mac Dowell, *Computers & Chemical Engineering*, 2017, **107**, 247-256.

5. R. Gross, P. Heptonstall, D. Anderson, T. Green, M. Leach and J. Skea, *UK Energy Research Centre*, 2006. http://www.ukerc.ac.uk/publications/the-costs-and-impacts-of-intermittency.html.

6. A. Boston and H. Thomas, *Energy Research Partnership. London*, 2015. http://erpuk.org/wp-content/uploads/2015/08/ERP-Flex-Man-Full-Report.pdf.

7. J. A. G. Brown, C. Eickhoff and D. J. Hanstock, 2014.

8. A. Brooke, D. Kendrick, A. Meeraus, R. Raman and R. Rasenthal, *GAMS Development Corporation: Washington, DC*, 1998.

9. A. M. Law, W. D. Kelton and W. D. Kelton, *Simulation modeling and analysis*, McGraw-Hill New York, 2007.

10. G. Wernet, C. Bauer, B. Steubing, J. Reinhard, E. Moreno-Ruiz and B. Weidema, *International Journal of Life Cycle Assessment*, 2016, **21**, 1218-1230.

11. B. PRé Consultants, *PRe Consultants BV, the Netherlands*, 2008. http://www.bockytech.com.tw/PDF-File/SimaPro7IntroductionToLCA.pdf.

12. EIA, *Annual Energy Outlook 2015 with projections to 2040*, 2015. https://www.eia.gov/outlooks/archive/aeo15/pdf/electricity_generation.pdf.

13. I. M. Algunaibet and W. Matar, *Energy Efficiency*, 2018, **11**, 1341-1358.

14. M. W. Ryberg, M. Owsianiak, K. Richardson and M. Z. Hauschild, *Ecological Indicators*, 2018, **88**, 250-262.

15. R. Frischknecht, N. Jungbluth, H.-J. Althaus, G. Doka, R. Dones, T. Heck, S. Hellweg, R. Hischier, T. Nemecek and G. Rebitzer, *The International Journal of Life Cycle Assessment*, 2005, **10**, 3-9.

16. E. Grubert and K. T. Sanders, *Environ Sci Technol*, 2018, **52**, 6695-6703.

17. W. Steffen, K. Richardson, J. Rockstrom, S. E. Cornell, I. Fetzer, E. M. Bennett, R. Biggs, S. R. Carpenter, W. de Vries, C. A. de Wit, C. Folke, D. Gerten, J. Heinke, G. M. Mace, L. M. Persson, V. Ramanathan, B. Reyers and S. Sorlin, *Science*, 2015, **347**, 1259855.

18. B. S. Koelbl, M. A. van den Broek, B. J. van Ruijven, A. P. C. Faaij and D. P. van Vuuren, *International Journal of Greenhouse Gas Control*, 2014, **27**, 81-102.

19. Lazard, *Lazard's Levelized Cost of Energy Analysis—Version 12.0*, Lazard, 2018. https://www.lazard.com/media/450784/lazards-levelized-cost-of-energy-version-120-vfinal.pdf.

20. EIA, *2016 Levelized Cost of New Generation Resources from the Annual Energy Outlook 2010*, 2010. https://www.eia.gov/outlooks/archive/aeo10/pdf/2016levelized_costs_aeo2010.pdf.

21. EIA, *Independent Statistics & Analysis*, Energy Information Administration, 2016. https://www.eia.gov/outlooks/ieo/pdf/0484(2016).pdf.

22. CEA, *Canada's electricity industry*, 2015. https://me.queensu.ca/Courses/MECH4301/Electricity101%20Report%20of%20Candian%20Electrical%20Association.pdf.

23. A. D. Cuellar and H. Herzog, *Energies*, 2015, **8**, 1701-1715.

24. NERC, *Potential Reliability Impacts of EPA's Proposed Clean Power Plan-Phase I*, North American Electric Reliability Corporation, 2015. https://www.nerc.com/pa/rapa/ra/reliability%20assessments%20dl/potential%20reliability%20impacts%20of%20epa%E2%80%99s%20proposed%20clean%20power%20plan%20-%20phase%20i.pdf.

25. W. Short, P. Sullivan, T. Mai, M. Mowers, C. Uriarte, N. Blair, D. Heimiller and A. Martinez, *Regional energy deployment system (ReEDS)*, National Renewable Energy Laboratory (NREL), Golden, CO., 2011.

26. A. Lopez, B. Roberts, D. Heimiller, N. Blair and G. Porro, *US renewable energy technical potentials: a GIS-based analysis*, NREL, 2012.

27. U. N. S. Division, National Accounts Statistics: Main Aggregates and Detailed Tables, 2017, https://unstats.un.org/unsd/nationalaccount/pubsDB.asp?pType=3, (accessed 08/11, 2018).

28. D. Iribarren, F. Petrakopoulou and J. Dufour, *Energy*, 2013, **50**, 477-485.

29. B. Corona, G. San Miguel and E. Cerrajero, *International Journal of Life Cycle Assessment*, 2014, **19**, 1264-1275.

30. F. Petrakopoulou, D. Iribarren and J. Dufour, *Greenhouse Gases: Science and Technology*, 2015, **5**, 268-276.

31. G. D. Oreggioni, B. Singh, F. Cherubini, G. Guest, C. Lausselet, M. Luberti, H. Ahn and A. H. Stromman, *International Journal of Greenhouse Gas Control*, 2017, **57**, 162-172.

32. W. B. Group, Population, https://data.worldbank.org/indicator/SP.POP.TOTL?locations=US, (accessed 08/11, 2018).

33. M. W. Ryberg, M. Owsianiak, J. Clavreul, C. Mueller, S. Sim, H. King and M. Z. Hauschild, *Sci Total Environ*, 2018, **634**, 1406-1416.

34. M. Fripp, *Environ Sci Technol*, 2012, **46**, 6371-6378.

35. U. S. A. C. o. Engineers, *Civil works construction cost index system*, 2011. https://www.publications.usace.army.mil.
